# Novel CDKs inhibitors for the treatment of solid tumour by simultaneously regulating the cell cycle and transcription control

**DOI:** 10.1080/14756366.2019.1705290

**Published:** 2020-01-03

**Authors:** Xin Wang, Kaiyuan Deng, Cheng Wang, Yao Li, Tianqi Wang, Zhi Huang, Yakun Ma, Peiqing Sun, Yi Shi, Shengyong Yang, Yan Fan, Rong Xiang

**Affiliations:** a Department of Medicinal Chemistry, School of Medicine, Nankai University, Tianjin, China; b 2011 Project Collaborative Innovation Center for Biotherapy of Ministry of Education, Tianjin, China; c State Key Laboratory of Medicinal Chemical Biology, Tianjin, China; d Department of Medical Oncology, Cancer Center, State Key Laboratory of Biotherapy, West China Hospital, Sichuan University, Chengdu, China; e Department of Cancer Biology, Wake Forest Comprehensive Cancer Center, Wake Forest School of Medicine, Winston-Salem, NC, USA

**Keywords:** CDK4, CDK9, inhibitor, cancer

## Abstract

A novel series of cyclin-dependent kinases (CDKs) inhibitors, which play critical roles in the cell cycle control and regulation of cell transcription, were synthesised. A systematic study of enzymatic and cellular assays led to the identification of compound **X22** with a nanomolar potency against CDK4 and CDK9 and potent antiproliferative activities against a panel of tumour cell lines. **X22** could induce cell cycle arrest and cell apoptosis in cancer cell lines. **X22** dose-dependently inhibits signalling pathways downstream of CDKs in cancer cells. *In vivo* antitumor activity assays, oral administration of **X22** led to significant tumour regression in mouse model without obvious toxicity. Superior anti-cancer efficacy *in vitro* and *in vivo* of **X22** demonstrated combined depletion of cell cycle and transcriptional CDK all contributed to antitumor activity. Taken together, concomitant inhibition of cell cycle and transcriptional CDK activities provided valuable guide for further structural optimisation.

## 1. Introduction

Cancer is a multigenetic disease with the hallmark that multiple signalling pathways aberration, which often require multiple therapeutic interventions^1^^,^^2^. Kinases mediate various cellular activities due to their critical roles in cellular signalling, such as proliferation, apoptosis, transcription, differentiation and so on^3^^,^^4^, which have been demonstrated as promising drug targets for the treatment of many diseases such as cancers^5–7^. Kinase inhibitors are widely employed in clinical oncology^8^^,^^9^. Simultaneous inhibition of different mechanisms using multi-kinase inhibitors could yield superior efficacy, such as synergy effects, avoiding drug resistance and so on^10^.

Out of numerous kinases, cyclin-dependent kinases (CDKs) are protein kinases involved in important cellular processes due to the complexity of their roles. They regulate the cell division, apoptosis, transcription and differentiation, which involved in a number of pathological conditions such as human cancer^11^^,^^12^. CDKs are divided into two groups based on their roles in cell cycle progression and transcription regulation^13^^,^^14^. The first group including CDK subtypes 1, 2, 4 and 6 mainly is involved in cell cycle and the second group including CDK subtypes 7, 8 and 9 are associated with transcription control^11^. CDK4/6 binding to cyclin D forming CDK4/6-cyclin D complex, is an important component of cell cycle activation and mediates the transition from G_1_ to S phase, where cells grow and synthesise proteins in preparation for DNA synthesis^15^. Among the transcriptional CDKs, CDK9 has attracted major interest of many groups. CDK9 forming heterodimeric complex with subunit cyclin T or cyclin K phosphorylates the COOH-terminal domain (CTD) of RNA polymerase II (RNAPII) to control the transcription progression^16^. As a component of a larger protein complex called positive transcription elongation factor b (P-TEFb), CDK9 stimulate transcription elongation of most protein coding genes^17^. Phosphorylation of RNAPII is regarded as a marker involved in a variety of human pathological conditions, such as cancer. Inhibition of CDK9 results in reduced levels of antiapoptotic proteins of cancer^18^^,^^19^.

Dysregulation of CDKs-cyclin pathway resulting in uncontrolled proliferation have been observed in various cancers^20–22^. Owing to the important role of CDKs in the control of cell division, numerous drugs targeting CDKs were designed and reported in the clinic^23^. Several selective CDK4/6 inhibitors have been reported in the recent years. Pfizer’s palbociclib represents the first CDK inhibitor approved by the US FDA in February 2015 to treat HR+/HER2-breast cancer^24^^,^^25^. Novartis’ ribociclib and Lilly’s abemaciclib also got a fast approval by FDA in March 2017 and September 2017, respectively, to treat HR+/HER2-breast cancer^26^^,^^27^. While as reported, both palbociclib and ribociclib as selective CDK4/6 inhibitors need to combine with letrozole for the treatment of breast cancer^28^^,^^29^. Abemaciclib with additional kinase activities, such as CDK1, CDK2 and CDK9 shows unique single-agent activities^30^^,^^31^. These studies indicate that monotherapy targeting of individual cell cycle CDKs may be insufficient for cancer therapy.

Simultaneous regulating the cell cycle and transcription control could provide superior anticancer efficacy^32–34^. In an effort to discover novel CDKs inhibitors with high inhibition potency against both cell cycle and transcriptional CDKs, we recently started a medicinal chemistry study based on ribociclib, a latest and selective CDK4 inhibitor and performed a rational drug design. According to the researches, many sulphur atom-containing compounds have immense importance in medicinal chemistry and exhibit anticancer activity^35^. Moreover, isothiocyanate plays a prominent role in the field of pharmaceuticals with anticancer effects in various cancer types^36^. Sulphur atom or isothiocyanate-containing derivatives are biologically active agents for drugs used for the treatment of cancer. We reasoned to introduce sulphur atom and isothiocyanate moiety based on pyrrolo-[2,3-d] pyrimidines-2-amine skeleton to proceed structural optimisation on R, L and linker regions (Supplementary Figure S1). In our study, we synthesised a series of novel compounds simultaneously mediating the cell cycle and transcription control of CDKs with high potent inhibition against solid tumour. Compound **X22** targeting CDK4 and CDK9 was discovered and exhibited potent antitumor efficacy *in vitro* and *in vivo*.

## 2. Materials and methods

### 2.1. General methods for chemistry

The commercially obtained chemicals were used directly without further purification. Solvents were purified and distilled following the standard procedures. All the reactions were monitored by thin-layer chromatography (TLC). The NMR spectra were taken on a Bruker AV-400 MHz spectrometer (400 MHz for ^1^H and 101 MHz for ^13 ^C) and chemical shifts were expressed in ppm downfield using tetramethylsilane as the internal standard. High-resolution mass spectra (HRMS) were performed on a VG ZAB-HS mass spectrometer under electron spray ionisation (ESI). All the derivatives for testing bioactivity were purified to >95% purity which was determined by HPLC analysis on a Shimadzu Prominence-i LC-2030C 3D system (column, InertSustain C18, 4.6 × 250 mm, 5 μM; mobile phase, gradient elution of methanol/H_2_O (90:10); low rate, 1.0 ml/min; UV wavelength, 190 − 800 nm; temperature, 40 °C; injection volume, 10 μL). The detailed synthesis of compounds is presented in Supplementary Material.

### 2.2. Molecular docking

Docking studies were finished by Discovery Studio 3.1 to explore the predicted binding modes of compound **X22** in CDK4, CDK6 (PDB code: 4EZ5) and CDK9 (PDB code: 4BCF) respectively. Hydrogen atoms were added by Gold (version 5.0). The images were created by PyMOL.

### 2.3. Cell lines and cell culture

All the human cancer cell lines were obtained from ATCC. Cells were cultured in RPMI-1640 (BioInd) medium according to the instructions from ATCC, with the medium containing 10% FBS (BioInd), 1% antibiotics (penicillin and streptomycin) at 37 °C in an atmosphere of 5% CO_2_.

### 2.4. Kinase inhibition assays

Kinase inhibition profiles were determined using KinaseProfiler services provided by Eurofins, and ATP concentrations used the Km of corresponding kinases.

### 2.5. Cytotoxicity assay

Cytotoxicity assay were conducted as described previously using a Cell Counting Kit-8 (CCK-8) assay (#CK04, Dojindo, Kumamoto, Japan)^37^.

### 2.6. Cell cycle assay

Cells were plated on six-well culture plates at a density of 5 × 10^5^ cells/mL and were treated with the indicated concentrations of **X22** or ribociclib for 12, 24, 36 or 48 h after they adherence. Cells were harvested and washed with phosphate buffered saline (PBS) for three times and then fixed with ice cold 75% ethanol overnight. The fixed cells were then washed with PBS and stained with propidium iodide (50 mg/mL) in the presence of RNase A (0.5 mg) for 30 min at 37 °C. The stained cells were then subjected to flow cytometry (Modfit, BD) for cell cycle analysis.

### 2.7. Annexin V-FITC/PI apoptosis assay

Cells at a density of 3 × 10^5^ cells/mL were seeded in six-well plates and treated with compounds at different concentrations for 48 h. The cells were then harvested and washed twice with cold PBS. Then the cells were subjected to an Annexin V/PI Apoptosis Detection kit (BD Biosciences) for staining according to manufacture’s instructions, and finally analysed by flow cytometry (Modfit, BD).

### 2.8. Western blotting

Protein extraction and western blotting methods were performed as described previously^37^. The antibodies used in this study including anti-β-actin (Santa Cruz; sc-47778), anti-p53 (CST; #9282), anti-phospho-p53^Ser15^ (CST; #9284), anti-Bax (CST; #2772), anti-Bcl-2 (BD; #51-6511GR), anti-Rb (CST; #9309), anti-phospho-Rb^Ser807/811^ (CST; #8516), anti-phospho-Rb^Ser780^ (CST; #9307), anti-RNA polymerase II CTD (Abcam; ab817), anti-RNA polymerase II CTD^phospho S5^ (Abcam; ab5131), anti-RNA polymerase II CTD^phospho S2^ (Abcam; ab5095).

### 2.9. *In vivo* assay

The experimental procedures of the animal study were proved by the Animal Care and Use Committee at Nankai University. 4T1 breast cancer cells (5 × 10^4^) were injected in the mammary fat pads of 6 − 8 weeks old female BALB/c mice. Once the tumours grew to a volume of approximately 100–150 mm^3^, they were placed into five treatment groups (*n* = 3, with a total tumour number of 15). The mice were treated daily for 18 days via oral gavage. Body weights and tumour size were determined every other day. Tumour measurements were found using a digital vernier calliper, and the volumes were determined using the following calculation: (short^2^) × long × 0.5. Experiments were performed under an approved IACUC protocol according to federal and institutional guidelines and regulations. Inhibition rate of tumour growth was calculated using the following formula: 100 × {1 − [(tumour volume_final_ − tumour volume_initial_) for **X22**-treated group]/[(tumour volume_final_ − tumour volume_initial_) for the vehicle-treated group]}.

### 2.10. Statistical analysis

Statistical analysis results were analysed values by GraphPad Prism version 6.0 software. For Student *t* test and ANOVA, *p* < 0.05 was considered statistically significant. Values were expressed as means ± SEM. Significance was determined by *χ^2^* test, others were determined by Student’s *t-*test. A value of *p* < 0.05 was used as the criterion for statistical significance. ***indicates significant difference with *p* < 0.001, **indicates *p* < 0.01, *indicates *p* < 0.05.

## 3. Results and discussion

**Scheme 1.**
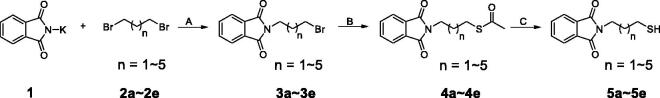
Synthesis of intermediates 5a–5e*^a^*. *^a^*Reagents and conditions: (A) Acetone, 70 °C, overnight-; (B) Potassium thioacetate (3.0 eq), THF, 75 °C, 5 h; (C) concentrated HCl, MeOH, 60 °C, 4 h.

**Scheme 2.**
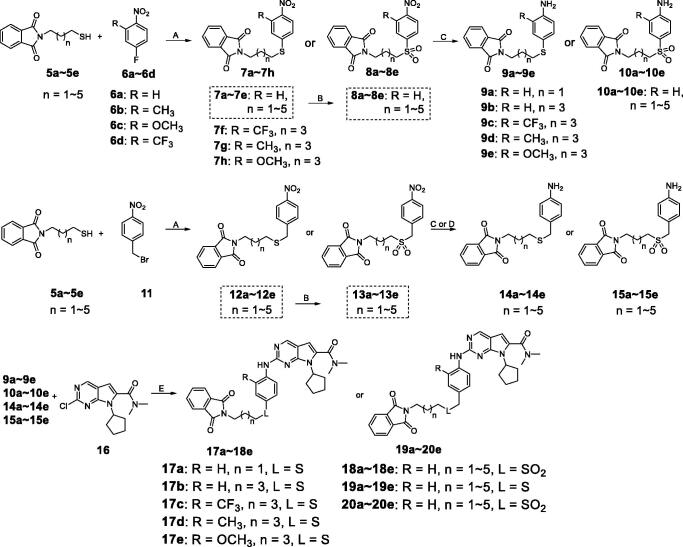
Synthesis of intermediates 17a–18e, 19a–20e*^a^*. *^a^*Reagents and conditions: (A) K_2_CO_3_ (2.0 eq), DMF, rt, 4.5 h; (B) m-CPBA (4.5 eq), DCM, 0 °C to rt, 2 h; (C) Fe/AcOH, MeOH, 60 °C, 5 h; (D) Zn, MeOH, rt, 1.5 h; (E) Pd(OAc)_2_ (0.10 eq), BINAP (0.06 eq), Cs_2_CO_3_ (2.0 eq), 1,4-dioxane, 105 °C, 7 h.

**Scheme 3.**
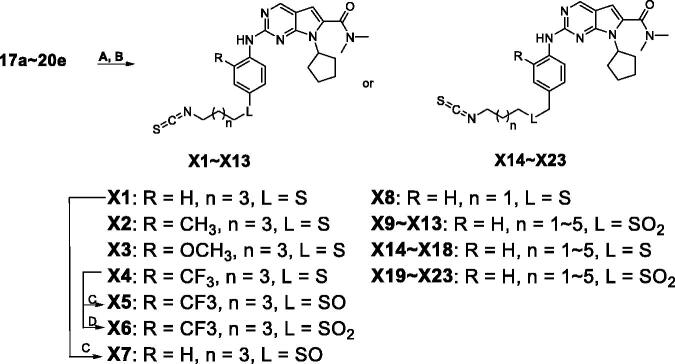
Synthesis of derivatives X1–X23*^a^*. *^a^*Reagents and conditions: (A) Hydrazine hydrate (6.0 eq), MeOH, rt, 3 h; (B) CS_2_ (20.0 eq), dicyclohexylcarbodiimide (DCC, 1.1 eq), THF, rt, overnight; (C) m-CPBA (1.5 eq) , DCM, −10 °C to 0 °C, 2 h; (D) m-CPBA (4.5 eq), DCM, 0 °C to rt, 2 h.

In our previous study, we found structural optimisation based on the pyrrolo-[2,3-*d*] pyrimidines-2-amine scaffold could improve the inhibitory activity against the cancer cell cycle as targeting CDK4/6 and transcription as targeting CDK9^38^. As the noteworthy antitumor functions of sulphur atom and isothiocyanate as mentioned above, we introduced these moieties to form molecules to investigate the structure–activity relationship. We changed the substituent at **R** position on the phenyl ring, as well as the functional group **L** and length of spacer between the isothiocyanato and phenyl ring. The pathways adopted for the synthesis of compounds **X1**–**X23** were carried out as shown in Schemes 1–3. The synthesis of the key intermediates **5a**–**5e** was depicted in Scheme 1. Firstly, refluxing the commercial obtained potassium phthalimide **1** with various dibromoalkane in acetone yielded the mono-bromides **3a**–**3e** bearing the phthalimide tail meeting the different length for SAR. Then, **3a**–**3e** were refluxed with potassium thioacetate (KSAc) in THF to afford thioacetates **4a**–**4e**, following acidic hydrolysis reaction in methanol to give the thiols **5a**–**5e**. In Scheme 2, prepared **5a**–**5e** were arylated by *p*-fluoronitrobenzene with various **R** substituents (**6a**–**6d**) to give the intermediates **7a**–**7h**. On the other hand, **5a**–**5e** reacted with 1-(bromomethyl)-4-nitrobenzene (**11**) in DMF under the alkaline condition of K_2_CO_3_ to obtain the **12a**–**12e**. Especially emphasised in dotted line cage, thioether intermediates **7a**–**7e** and **12a**–**12e**, were oxidised absolutely to corresponding sulphone **8a**–**8e** and **13a**–**13e**. Subsequently, nitro group in intermediates **7a**–**8e** and **12a**–**13e** need to be reduced to primary amine using either iron powder activated by acetic acid or zinc powder (**9a**–**9e**, **10a**–**10e**, **14a**–**14e**, **15a**–**15e**). After that, all the primary amine intermediates described above underwent Buchwald–Hartwig coupling reaction with commercially available **16** by catalytic amount of palladium acetate to create the key intermediates **17a**–**18e** and **19a**–**20e**. As shown in Scheme 3, the derivatives except **X5**–**X7** were produced via two successive steps without intermediate purification. **17a**–**20e** first were refluxed with 80% hydrazine hydrate in methanol to deprotect, following reacting with carbon disulphide and *N*,*N*′-dicylohexylcarbodiimide to afford the isothiocyanates derivatives **X1**–**X4** and **X8**–**X23**. Finally, sulphone derivative **X6** was obtained by oxidising **X4** completely with an excess of m-CPBA. Sulfoxide derivatives **X5** and **X7** were obtained through incomplete oxidation from **X4** and **X1**, respectively, which were accomplished through controlling strictly the amount of 1.1 equiv m-CPBA.

The results of the enzymatic-inhibition assays are listed in Tables 1 and 2 with ribociclib as the positive control. We expected to explore an optimisation strategy for improving the inhibitory activity against cell cycle as targeting on CDK4/6 and simultaneously targeting CDK9 for cell transcription. The structure–activity relationships were discussed based on the optimisation at the R, L substituents and the carbon tail length (shown as Formula I and II in Table 1).

**Table 1.** Structure and enzymatic inhibition activity evaluation of compounds **X1–X23**.^a^
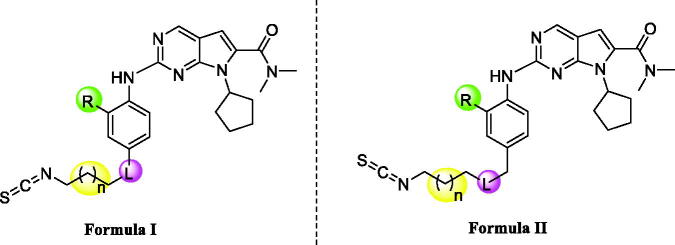

	Structure				Inhibition (%) at 1 μM		
Compds.	Formula	R	L	n	CDK4	CDK6	CDK9
Ribociclib	–	–	–	–	101 ± 0	85 ± 1.2	84 ± 0
**X1**	I	H	S	3	35 ± 0.5	63 ± 1.2	76 ± 0
**X2**	I	CH_3_	S	3	29 ± 2.1	17 ± 1.2	80 ± 0
**X3**	I	OCH_3_	S	3	8 ± 2.1	5 ± 0.8	33 ± 2.5
**X4**	I	CF_3_	S	3	9 ± 1.6	8 ± 3.3	41 ± 0.8
**X5**	I	CF_3_	SO	3	91 ± 0.8	48 ± 1.2	97 ± 0.4
**X6**	I	CF_3_	SO_2_	3	35 ± 2.4	14 ± 1.2	75 ± 0.4
**X7**	I	H	SO	3	99 ± 0	87 ± 1.6	99 ± 0
**X8**	I	H	S	1	70 ± 2.4	64 ± 0.8	97 ± 0.4
**X9**	I	H	SO_2_	1	94 ± 1.6	92 ± 1.2	97 ± 0
**X10**	I	H	SO_2_	2	90 ± 0.8	88 ± 0.8	98 ± 0.4
**X11**	I	H	SO_2_	3	89 ± 0.4	82 ± 0.8	98 ± 0.4
**X12**	I	H	SO_2_	4	83 ± 0	75 ± 0.4	97 ± 0.4
**X13**	I	H	SO_2_	5	74 ± 0	68 ± 0.4	96 ± 0.4
**X14**	II	H	S	1	95 ± 0	86 ± 1.2	97 ± 0.4
**X15**	II	H	S	2	83 ± 0.8	76 ± 0.8	96 ± 0
**X16**	II	H	S	3	71 ± 0.8	73 ± 0	94 ± 0.4
**X17**	II	H	S	4	61 ± 2.1	52 ± 0.4	94 ± 1.6
**X18**	II	H	S	5	82 ± 1.2	52 ± 0.8	95 ± 0.4
**X19**	II	H	SO_2_	1	98 ± 0	94 ± 0.8	98 ± 0.4
**X20**	II	H	SO_2_	2	99 ± 0.4	94 ± 0.4	98 ± 0.4
**X21**	II	H	SO_2_	3	98 ± 0.4	93 ± 0	98 ± 0
**X22**	II	H	SO_2_	4	97 ± 0.4	91 ± 0	98 ± 0
**X23**	II	H	SO_2_	5	95 ± 0.5	77 ± 1.6	97 ± 0

^a^ Inhibition activities were determined using the KinaseProfiler of Eurofins. The data represent the mean values of two independent experiments.

**Table 2.** Enzymatic inhibition activity evaluation of selected compounds.^a^

	IC_50_ (nM)				IC_50_ (nM)		
Compds.	CDK4	CDK6	CDK9	Compds.	CDK4	CDK6	CDK9
Ribociclib	13 ± 1.3	71 ± 14.1	197 ± 14.8	**X15**	–	–	29 ± 1.4
**X5**	331 ± 33.3	–	34 ± 5.3	**X16**	–	–	34 ± 1.4
**X7**	26 ± 1.1	–	7 ± 0.4	**X17**	–	–	55 ± 2.2
**X8**	–	–	16	**X18**	–	–	42 ± 1.6
**X9**	43 ± 2.6	101 ± 4.7	4 ± 0.4	**X19**	17 ± 0.7	55 ± 1.6	6 ± 0.4
**X10**	90 ± 10.5	–	7 ± 0.8	**X20**	21 ± 1.4	42 ± 5.4	7 ± 1.0
**X11**	–	–	8 ± 0.8	**X21**	24 ± 0.9	78 ± 13.4	7 ± 0.6
**X12**	–	–	13 ± 1.8	**X22**	30 ± 3.9	126 ± 20.6	10 ± 1.3
**X13**	–	–	24 ± 3.1	**X23**	75 ± 10.0	–	14 ± 0.9
**X14**	79 ± 1.3	–	16 ± 2.8				

^a^ IC_50_ values were determined using the KinaseProfiler of Eurofins. The data represent the mean values of two independent experiments.

Firstly, we explored the **R** position with various substituents, such as H, CH_3_, OCH_3_, CF_3_ (**X1**–**X4**, Table 1). The results showed that H and CH_3_ could only maintain the potent against CDK9 compared with ribociclib, while the OCH_3_ and CF_3_ groups were not tolerable at all because of the cliff loss potency on all the three kinases. In total, all the optimisation on **R** position would decrease enzymatic inhibitory activity on different levels, especially for CDK4/6. We presumed whether the joint position **L** between the isothiocyanato tail and phenyl ring affected the activity. Thus, **X4** as a candidate for its poor potency on activity was selected to be oxidised to **X5** and **X6** structurally characterised as sulfoxide and sulphone, all of which were tested together to confirm the importance of the joint position **L** as shown in Table 1. As expected, **X5**–**X6** displayed more notable increasing inhibitory activity than **X4** against all of three kinases. Oxidation state of sulphur at the joint position **L** might be superior than sulphur, that was also be confirmed in the example of **X1** versus **X7** (Table 1). Further, the derivatives bearing oxidation state of sulphur were tested the IC_50_ value against CDK4 and CDK9 due to the high inhibition at 1 micromolar concentration (Table 2). Compared with ribociclib, **X7** exhibited excellent potency against CDK4/9 (IC_50_: 7 and 26 nM, respectively). At the same time, a derivative (**X8**) with different space between isothiocyanato and phenyl ring was synthesised to determine the changing the activity produced by the length of the tail. The results indicated that the length of the tail could cause changing on the enzymatic inhibitory activity, but still need to be explored the rules of the changing in detail.

Based on preliminary research, we then adopted the H at **R** position to design several derivates (**X9**–**X13**, Table 1) to focus on the effect of the sulphur and its oxidation state at **L** position and length of the tail on the enzyme test. Obviously, **X9** with sulphone enhanced the inhibition against the three kinases, compared with **X8**. In the example of compound **X9**–**X13**, the inhibition against CDK9 was keeping at a relative high level, but the efficacy activity on CDK4/6 was declined with the increasing space between the isothiocyanato and sulphone structurally.

In view of the importance of the **L** position and the length of the tail, we attempt to insert a methylene between the **L** position and the phenyl leading to derivatives **X14**–**X23** (Table 1). Excitingly, **X19**–**X23** were proved high inhibition on all three kinases as well as no obvious difference for the enzymatic activity but **X23**. We summarised the structure–activity relationships as shown in Supplementary Figure S2.

Since our goal here is to discover CDKs inhibitors with cell cycle arrest and transcription blockade activities having high potency for cancer treatment, we screened compounds exhibiting better kinase inhibitory to do the cytotoxicity assays. The selected compounds, including **X7**, **X19**, **X20** and **X22** were carried out using standard CCK8 assay and the results are shown in Table 3. **X8** and **X12** with selective CDK9 inhibition and ribociclib as CDK4 inhibitor were assess the cancer cell inhibition assays as control. IC_50_ values were detected on compounds against breast cancer cell lines (4T1, T47D and MCF7) and lung cancer cell lines (A549, H1299 and H460). The best tumour cell potency of these analogues was obtained for molecule **X22**, which was also rational inhibitor of CDK9 with IC_50_ value of 10 nM and CDK4 with IC_50_ value of 30 nM. The CDK4 inhibitor ribociclib alone was not potent in these solid tumour cells. **X22** displayed higher inhibition potency than selective CDK4 inhibitor ribociclib and selective CDK9 inhibitor **X8** and **X12**. This result demonstrated that dual-target compound had more efficient inhibition at the cellular level. As the results of enzymatic and antitumor activity in cell lines showed that **X22** is the best in this class, we performed further in-depth *in vitro* and *in vivo* biological studies using this compound.

**Table 3.** *In vitro* cell growth inhibition (IC_50_) of selected compounds against multiple cancer cell lines.^a^

	IC_50_ (μM)					
Cell lines	4T1	T-47D	MCF7	A549	H460	H1299
Ribociclib	>10	6.23 ± 4.04	>10	7.46 ± 3.06	>10	5.46 ± 2.65
**X8**	1.46 ± 0.34	0.41 ± 0.10	1.33 ± 0.15	3.07 ± 0.11	3.22 ± 0.22	3.79 ± 0.07
**X12**	0.46 ± 0.07	0.16 ± 0.02	0.57 ± 0.24	1.57 ± 0.07	2.79 ± 0.32	1.31 ± 0.06
**X7**	0.41 ± 0.10	0.10 ± 0.02	0.23 ± 0.04	0.67 ± 0.03	0.92 ± 0.15	1.15 ± 0.10
**X19**	0.69 ± 0.14	0.72 ± 0.03	0.16 ± 0.06	1.46 ± 0.28	1.45 ± 0.10	0.60 ± 0.04
**X20**	0.26 ± 0.19	0.12 ± 0.01	0.12 ± 0.07	0.38 ± 0.02	1.50 ± 0.39	0.60 ± 0.24
**X22**	0.083 ± 0.34	0.11 ± 0.01	0.11 ± 0.07	0.21 ± 0.07	1.80 ± 0.34	0.57 ± 0.07

^a^ The IC_50_ values are shown in the forms. The cytotoxic effect of compounds was assayed using CCK-8 assay with 72 h incubation. Data are from three independent experiments.

**Figure 1.**
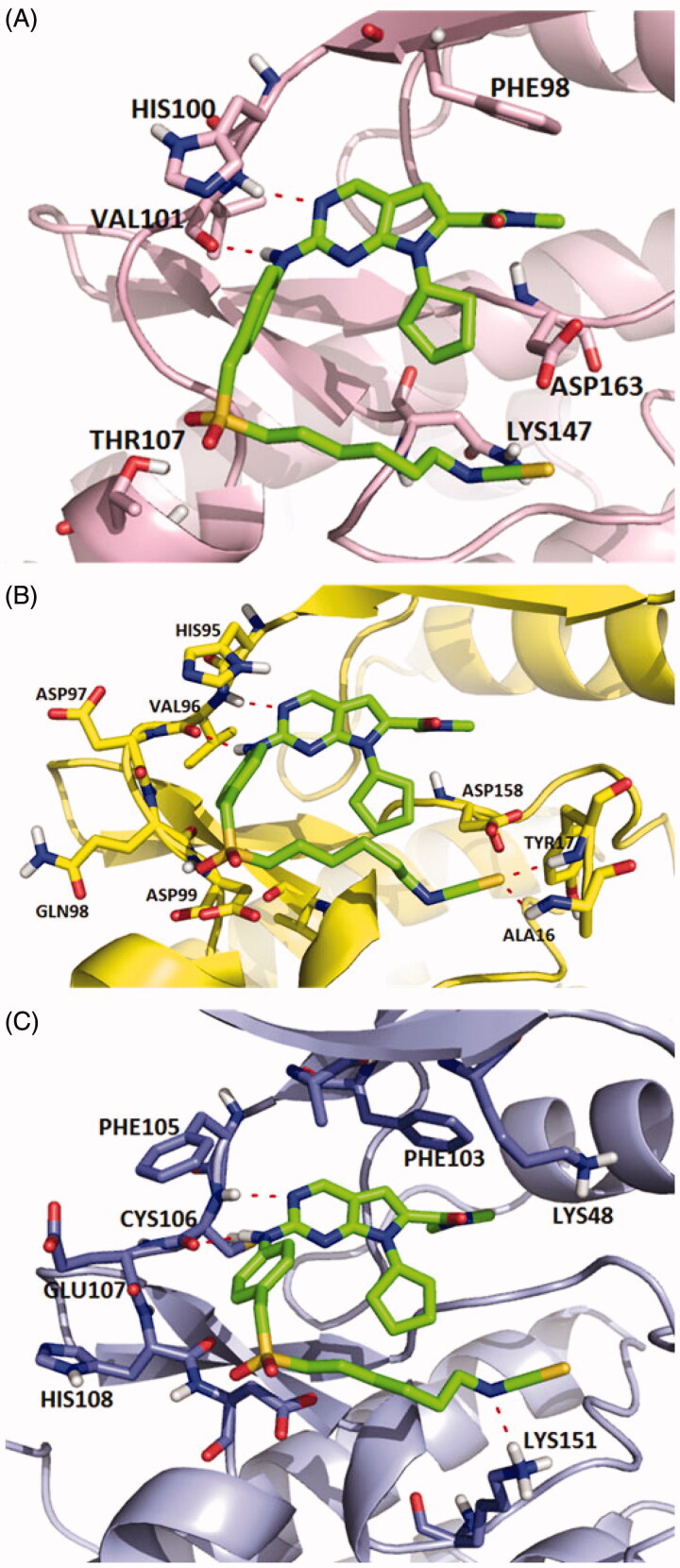
Representation of the predicted binding modes of compound X22 with CDKs kinase domain. X22 is shown in green. Hydrogen bonds are shown in red. (A) Proposed binding mode of compound X22 with CDK6 (PDB code: 4EZ5), CDK6 backbone is shown in light pink. (B) Proposed binding mode of compound X22 with CDK4, which employed CDK6 (PDB entry: 4EZ5) as the template for homology modelling, CDK4 backbone is shown in yellow. (C) Proposed binding mode of compound X22 with CDK9 (PDB code: 4BCF), CDK9 backbone is shown in light blue.

Molecular docking studies were carried out to investigate the binding modes of compound **X22**. **X22** is a potent CDK4 and CDK9 inhibitor with IC_50_ value of 30 and 10 nM, respectively, and also has weak inhibition activity against CDK6 with IC_50_ value of 126 nM. As shown in Figure 1(A), **X22** binds to the ATP-binding sites of CDK6 with two key hydrogen bonds formed by aminopyrimidine and the backbone VAL101 residue, which retained the binding mode of the scaffold of ribociclib and CDK6 as reported. Given no 3D CDK4-ligand was reported, the structure of CDK6-ligand complex (PDB code: 4EZ5) was used as a template for homology modelling to generate the 3D structure of CDK4-ligand (Figure 1(B)). Importantly, two additional hydrogen bonds were formed because of the hydrophobic side chain containing isothiocyanate. The sulphur atom formed two hydrogen bonds with the backbone of ALA16 and TYR17 at the front of the ATP binding site of CDK4, respectively, which might explain the better inhibitory activity of the compound **X22** against CDK4 (IC_50_ = 30 nM) than CDK6 (IC_50_=126 nM). In Figure 1(C), the 2-aminopyrimidine formed two hydrogen bonds with the CYS106 residue of **X22** in CDK9 (VAL101 in CDK6, VAL in CDK4). As well, the nitrogen atom formed a key hydrogen-bond with the residue of the LYS151 at the front of the ATP binding pocket. At the back of the ATP binding site, the pyrrolo-[2,3-*d*]-pyrimidine scaffold exploited the hydrophobic region close to the gatekeeper residue to form a favourable π–π interaction in all three kinases region.

**Figure 2.**
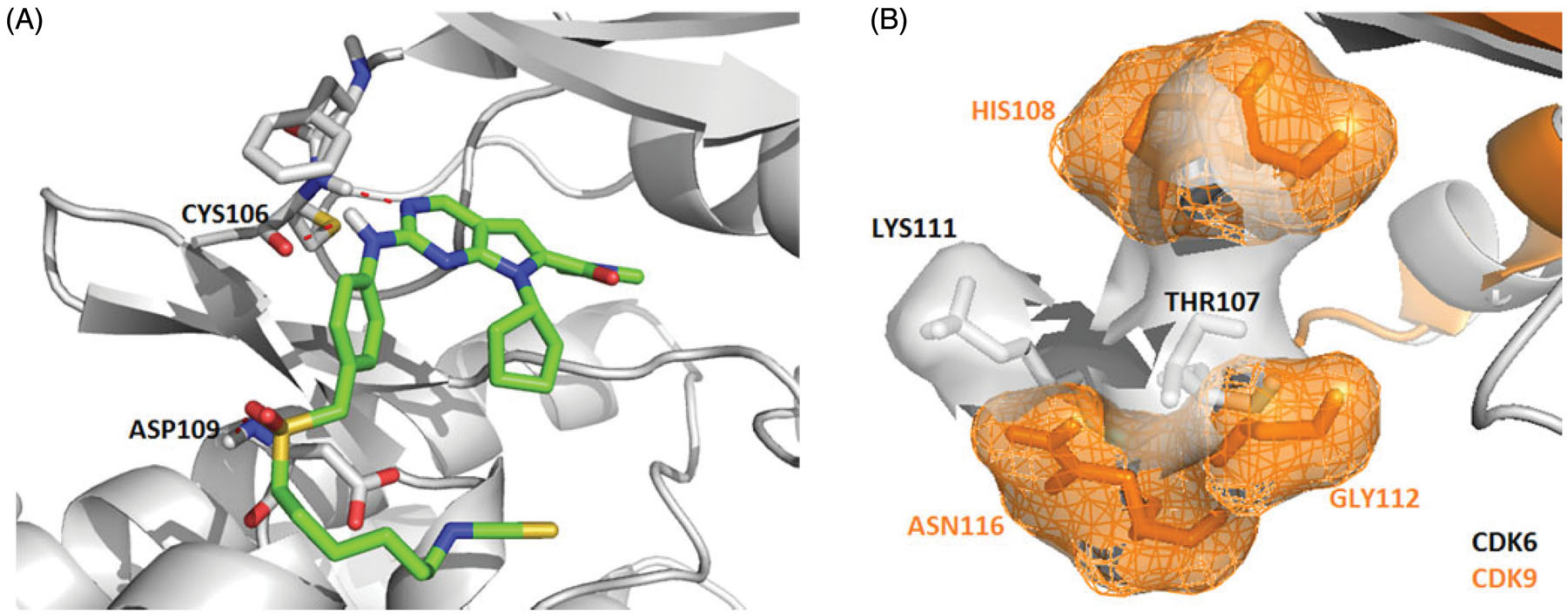
(A) Representation of the predicted binding mode of compound X22 with CDK9 kinase domain (PDB code: 4BCF). CDK9 backbone is shown in grey. X22 is shown in green. Hydrogen bonds are shown in red. (B) Representation of the hydrophobic pocket difference at the front of the ATP-binding site by overlaying CDK6 and CDK9. CDK6 is shown in grey. CDK9 is shown in orange.

Interestingly, a hydrogen-bond was formed between the oxygen of the sulphonyl of **X22** with the backbone of the ASP109 in another docking configuration (Figure 2(A)). At the front of the ATP binding pocket in CDK9, GLY112 possessing the smallest residue makes this hydrophobic site accommodate flexible side chain like compound **X22**, while THR107 at the same position in CDK6 shows a little crowded for flexible group (Figure 2(B)). The much bigger hydrophobic site permit the compound **X22** exits in multiple configurations docking CDK9 forming hydrogen bond for binding tightly. The difference might offer an explanation the stronger inhibitory potency compound **X22** against CDK9 (IC_50_= 10 nM). Thus, **X22** as an ATP-competitive inhibitor potently binds in the ATP pockets of CDKs kinases.

**Figure 3.**
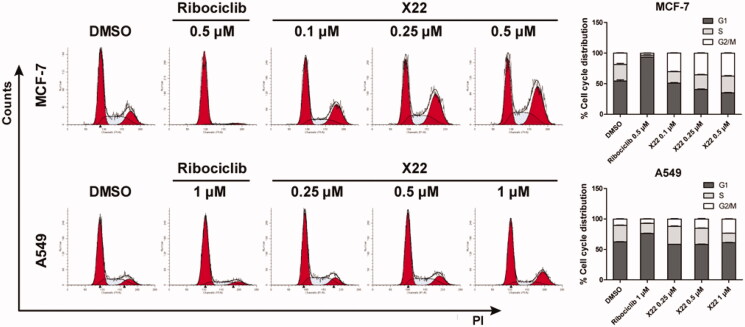
X22 induced G_2_/M phase arrest in breast and lung cancer cells. Cells were harvested after treatment with different concentrations of X22 or ribociclib for 24 h. Cells were fixed by 70% ethanol and stained with propidium iodide. Data are represented as histogram on the left and the percentage of cell cycle distribution are shown on the right. The assays were performed in triplicate.

Given the strong inhibitory ability of **X22** in MCF7 and A549 cells, we examined the effect of **X22** on the cell cycle. Cells were treated with compound **X22**, ribociclib and DMSO for 24 h. Representative flow cytometry patterns are shown in Figure 3. The results showed that **X22** significantly blocked the cell cycle at G_2_/M phase in a dose-dependent manner accompanied by decreases in S phase in both cell lines, as compared to the cells incubated with DMSO. Ribociclib mainly blocked the cell cycle at G_1_ phase (Figure 3). We also detected cell kinetics treated with **X22**, ribociclib and DMSO for 12, 36 and 48 h (Supplementary Figure S3). These results confirmed that **X22** induced the cell cycle arrest, which are consistent with our observations above^39^. Compared with CDK4 depletion alone, combined depletion CDK4 and CDK9 induced substantial G_2_/M arrest. These data indicated that **X22** could induce G_2_/M phase arrest of the cell cycle in both breast and lung cancer cells.

As reported^40^^,^^41^, CDK4/6 preferentially bind D-type cyclins to form complexes. Activations of these complexes are responsible for the phosphorylation of retinoblastoma protein (Rb), which allows cell cycle to proceed from G_1_ to S phase and further results in cell proliferation. In contrast, CDK9 is not a typical Cdc-2-like kinase and does not participate in cell cycle regulation. It can form the P-TEFb complex, which is capable of phosphorylating the CTD of the largest subunit of RNAPII and regulate the RNA transcription elongation.

**Figure 4.**
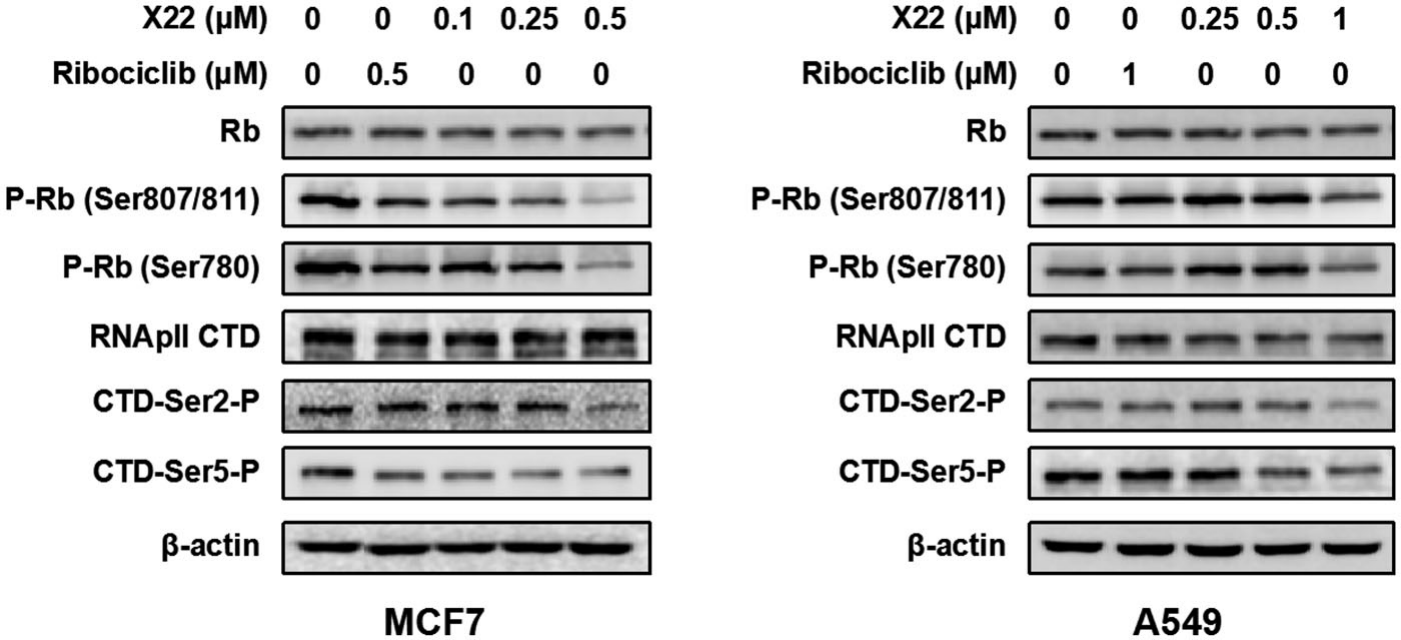
X22 suppressed the downstream signalling proteins of CDK4/9 in breast and lung cancer cells. Cells were incubated with the indicated concentrations of X22 or ribociclib for 24 h. Proteins were extracted and analysed by western blotting.

To further verify the inhibitory effect of compound **X22** on CDKs, western blotting analysis was performed. We found that **X22** treatment significantly suppressed the phosphorylation of Rb at CDK4/6 specific site Ser 780 and CDK9 specific sites Ser 807/811 in MCF7 and A549 cells respectively, which confirmed that **X22** targeted on CDKs (Figure 4). We also detected the level of phosphorylated Ser2 (Ser2-P), a well-established cellular target of CDK9 on the RNAPII CTD during transcription elongation. Compared to cells treated with DMSO, **X22** effectively inhibited the phosphorylation of Ser2 and Ser5 at 0.5 µM in MCF7 cells and 1 µM in A549 cells (Figure 4). Taken together, the results above revealed that **X22** could specifically target CDKs, thus further regulating their downstream signalling proteins in cancer cells.

**Figure 5.**
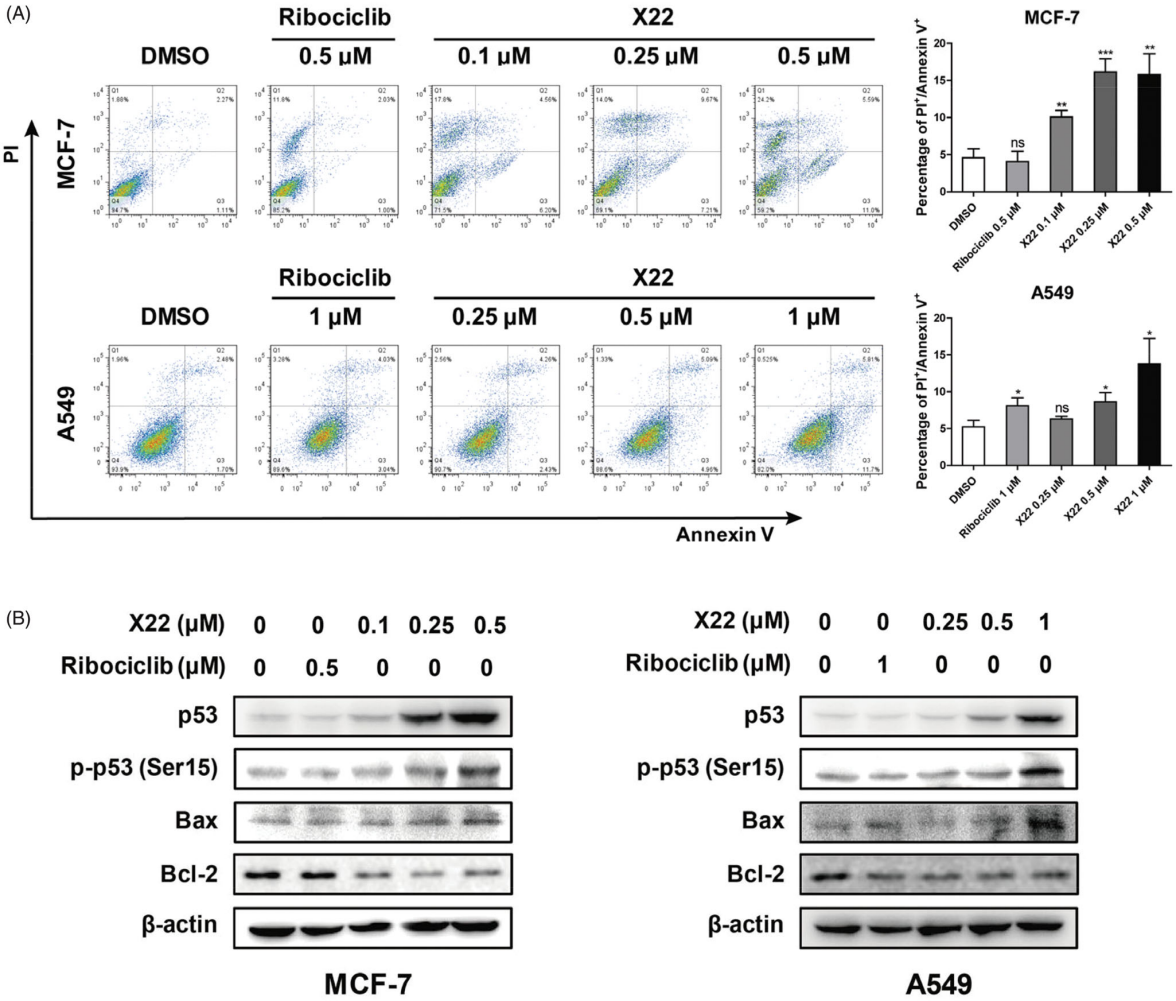
X22 induced cell apoptosis in breast and lung cancer cells. (A) Cells were seeded in six-well plates and treated with the indicated concentrations of X22 or ribociclib for 48 h. Cells were then stained with the AnnexinV-FITC Apoptosis Detection Kit, followed by flow cytometry analysis. Quantitative data are expressed as mean ± SD of the percentages of apoptotic cells from three independent experiments. (B) Expressions of p53, phospho-p53 (Ser15), Bax, Bcl-2 in MCF-7 and A549 cells were detected by western blotting after the treatment with the indicated concentrations of ribociclib or X22 for 48 h. *p < 0.05 versus DMSO; **p < 0.01 versus DMSO; ***p < 0.001 versus DMSO.

We next analysed whether **X22** treatment promoted cell apoptosis by Annexin V-FITC/PI staining. After 48 h incubation, **X22** dose-dependently increased the proportion of apoptotic cells (Q2 late apoptotic and Q3 early apoptotic) in A549 cells, compared to the cells treated with DMSO or ribociclib. Similar results were also observed in MCF7 cells. **X22** at 0.1 to 0.5 µM increased the percentages of apoptotic cells by approximately 2.2 to 3.5-fold change, compared to DMSO-treated cells (Figure 5(A)). To further confirm the apoptosis induction of compound **X22**, we examined the expression of relevant proteins by Western blot. Cells were treated with or without compound **X22** and control for 48 h and then lysed and analysed. Given the crucial role of p53 in regulating cell apoptosis, we detected whether compound **X22** induced apoptosis depending on the expression of p53 and its downstream targets. As illustrated in Figure 5(B), **X22** treatment remarkably increased the expressions of p53 and promoted the phosphorylation of p53 at Ser15. We also demonstrated that **X22**-induced apoptosis in solid tumour cells, which reduced expression of the anti-apoptotic protein Bcl-2 and enhanced expression of Bax. These findings indicated that compound **X22**-induced apoptosis is associated with upregulating pro-apoptotic protein p53 and Bax and downregulating anti-apoptotic protein Bcl-2 expression. Collectively, **X22** may induce cell apoptosis through p53/Bax/Bcl-2 pathway in breast and lung cancer cells.

**Figure 6.**
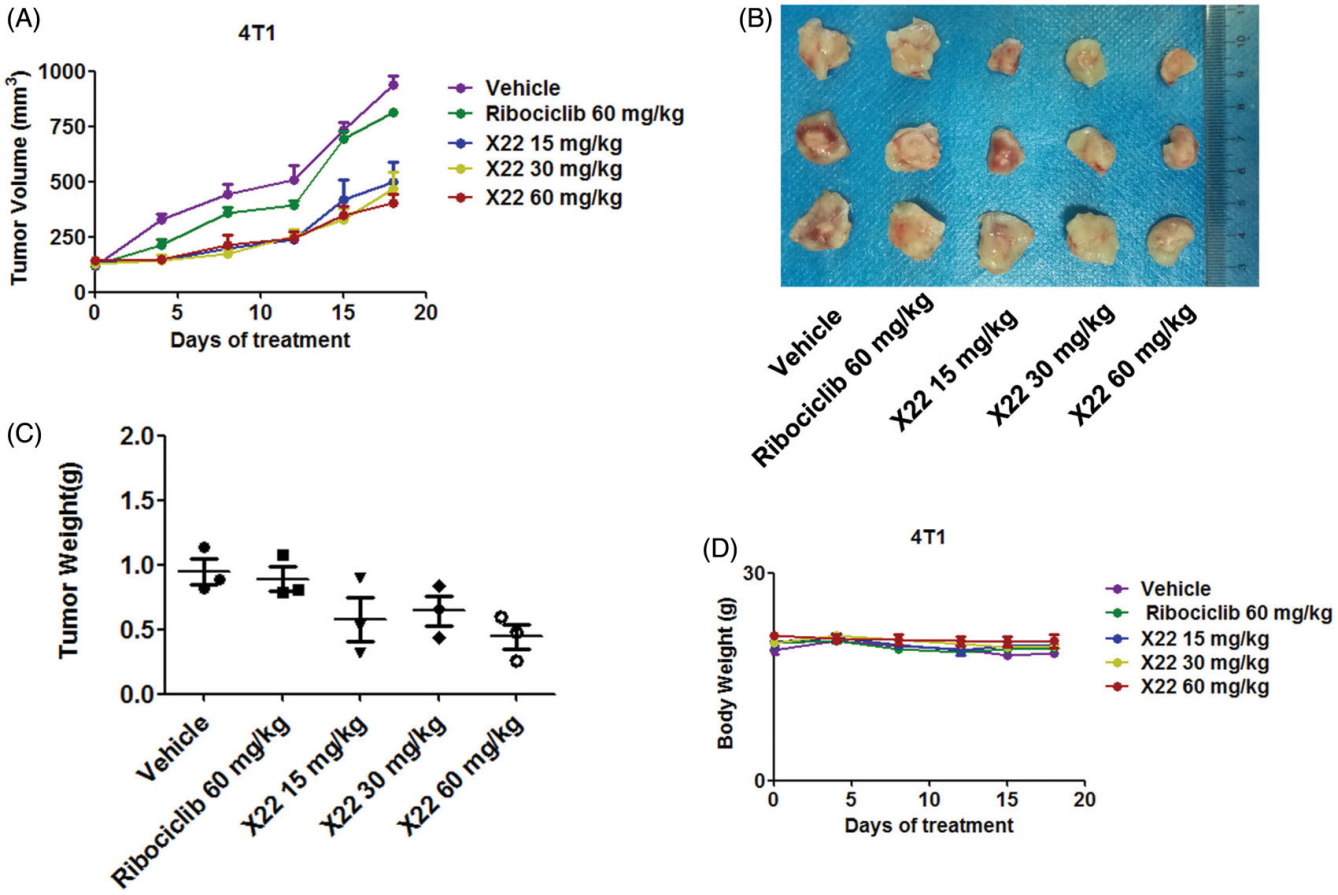
X22 significantly inhibited growth of breast cancer in homograft mouse models. (A) Tumour growth curve of 4T1 tumour-bearing mice in response to different treatments. (B) Photographs of 4T1 homograft tumours. Mice were sacrificed 18 days after tumour implantation. (C) The average tumour weight of excised tumours at day 18. (D) Body weight curves of BALB/c mice in each group after injection.

To better assess whether **X22** could efficiently suppress tumour growth *in vivo*, homograft tumour models were established using 4T1 cells. Oral treatment with **X22** significantly suppressed the tumour growth (Figure 6(A)). Representative photographs of excised tumours at day 18 were shown in Figure 6(B). **X22** treatment with 15 or 30 or 60 mg/kg substantially inhibited the tumour growth with tumour inhibition rates of 51.8 ± 17.5%, 65.4 ± 11.7% and 70.3 ± 4.4%, respectively. In contrast, tumour volume in ribociclib-treated group decreased slightly compared with vehicle group. Tumour growth inhibitions of 17.2 ± 5.2% were observed at doses of 60 mg/kg of ribociclib. The potencies of ribociclib are relatively weaker than those of **X22**. The average tumour weight of excised tumours in **X22** groups was obviously lighter than those in other groups (Figure 6(C)). A preliminary toxicity evaluation for **X22** was also carried out. Mouse weights were monitored twice per week over 18 days. There was no significant difference among the five groups of mice (Figure 6(D)). No obvious signs of toxicity were observed in the **X22**-treated groups, indicating that compound **X22** is well tolerated *in vivo*.

## 4. Conclusions

To date, selective CDK inhibitors have produced only modest activity against solid tumours. In this article, we discovered and studied the medicinal chemistry of multi-CDKs inhibitors to assess their relative activities against the cell cycle and transcriptional CDKs. Several compounds inhibited CDK4 and CDK9 at low nanomolar levels and exhibited good antiproliferative activities in a panel of tumour cells. The most potent analogue **X22** potently inhibited CDK4 and CDK9 with IC_50_ values of 30 and 10 nM, respectively, which showed marked antitumor activity. In *in vitro* cellar assays, **X22** was capable of blocking the cell cycle at G_2_/M phase resulting in decreased S-phase populations. Compared with CDK4 depletion alone, combined depletion induced apoptosis in cancer cells in a dose-dependent manner. Western blot assays confirmed substantial apoptosis after **X22**-induced CDK4 and CDK9 depletion. Meanwhile, **X22** reduced RNA polymerase II expression and CTD phosphorylation. In *in vivo* assay, oral administration of compound **X22** once-daily at 15, 30 or 60 mg/kg for 18 days led to tumour regression without obvious toxicity. Thus, reduced CDK4 activity in concert with depleted CDK9 activity may enhance the antiproliferative effects. This study suggest combined depletion of cell cycle and transcriptional CDK activities may be worthy of clinical further development for solid tumour therapy.

## Supplementary Material

Supplemental Material
