# Supplementary material for: Novel CDKs inhibitors for the treatment of solid tumour by simultaneously regulating the cell cycle and transcription control
Source: J Enzyme Inhib Med Chem. 2020 Jan 3;35(1):414–23. doi: 10.1080/14756366.2019.1705290 (PMC6968521; doi:10.1080/14756366.2019.1705290)
Supplement: Supplemental Material [file IENZ_A_1705290_SM8879.pdf]

Novel CDKs inhibitors for the treatment of solid tumor by simultaneously regulating the cell cycle and transcription control

Xin Wang,[⊥a] Kaiyuan Deng,[⊥a] Cheng Wang,[a] Yao Li,[a] Tianqi Wang,[a] Zhi Huang,[a] Yakun Ma,[a] Peiqing Sun,[a, e] Yi Shi,[a, b] Shengyong Yang,[d] Yan Fan*[a,b] and Rong Xiang*[a, b, c]

[a] Department of Medicinal Chemistry, School of Medicine, Nankai University, 94 Weijin Road, Tianjin 300071, China; [b] 2011 Project Collaborative Innovation Center for Biotherapy of Ministry of Education, 94 Weijin Road, Tianjin 300071, China; [c] State Key Laboratory of Medicinal Chemical Biology, 94 Weijin Road, Tianjin 300071, China; [d] Medical Oncology, Cancer Center, State Key Laboratory of Biotherapy, West China Hospital, Sichuan University, Chengdu, China; [e] Department of Cancer Biology, Wake Forest Comprehensive Cancer Center, Wake Forest School of Medicine, Winston-Salem, NC, USA.

**CONTACT *Yan Fan and *Rong Xiang. Department of Medicinal Chemistry, School of Medicine, Nankai University, 94 Weijin Road, Tianjin, China. These authors contribute equally.*

Author Contributions: [#]Xin Wang and Kaiyuan Deng are contributed equally to this work.

Figure S1. The region of structure optimization for CDKs inhibitor......................3

Figure S2. The structure-activity relationships for CDKs inhibitor....................... 4

Figure S3. X22 induces G2/M phase arrest in breast cancer and NSCLC cells ... 5

General Methods for Chemistry... 6

Copy of 1H- and 13C-NMR Spectra for Compound X22...................................... 46

Copy of HRMS Spectra for Compound X22... 47

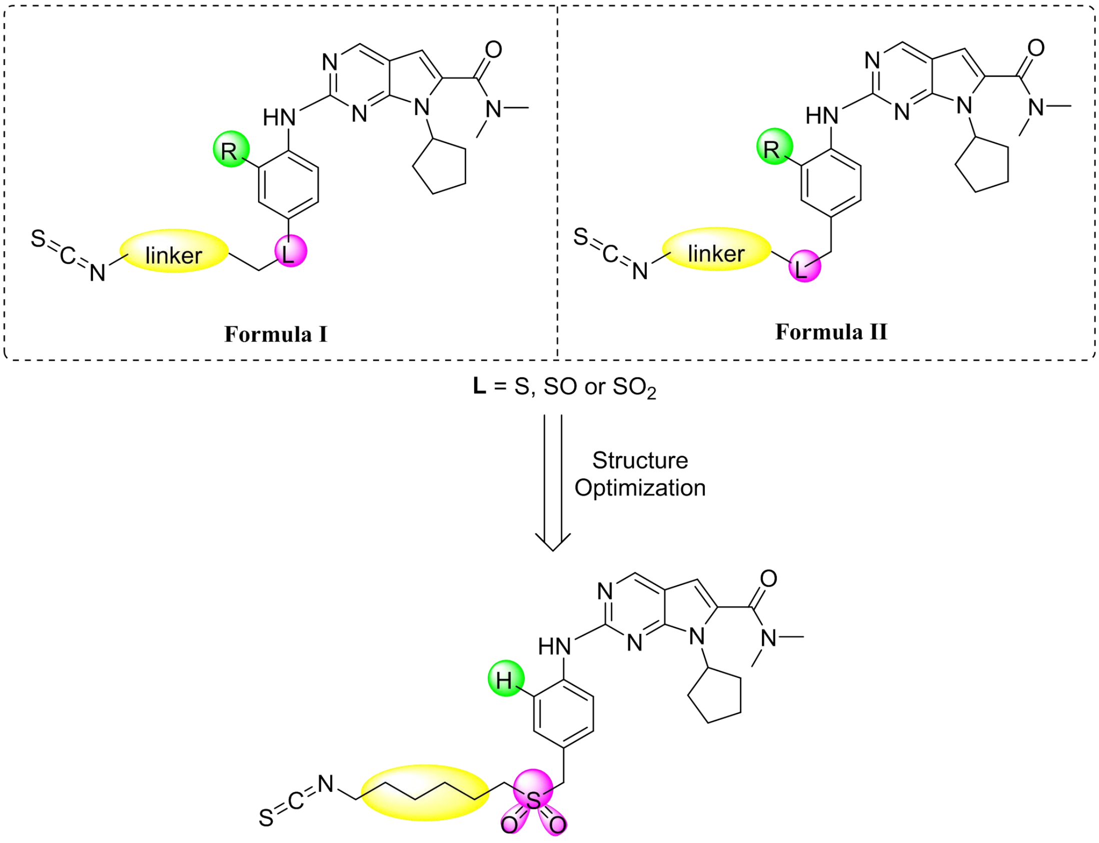

X22

CDK9 IC$_{50}$ = 10 nM   MCF7 IC$_{50}$ = 110 nM

CDK4 IC$_{50}$ = 30 nM   A549 IC$_{50}$ = 151 nM

Figure S1. The region of structure optimization for CDKs inhibitor.

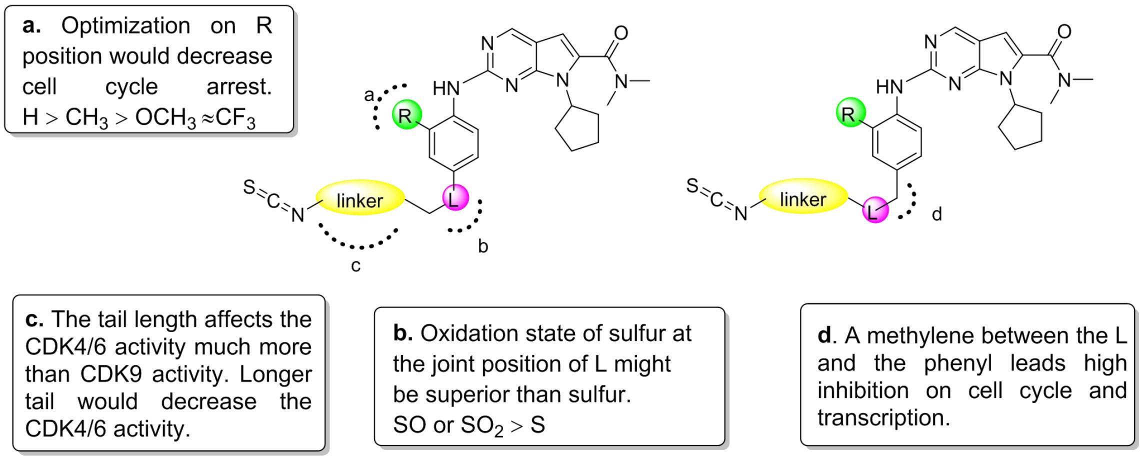

Figure S2. The structure-activity relationships for CDKs inhibitor.

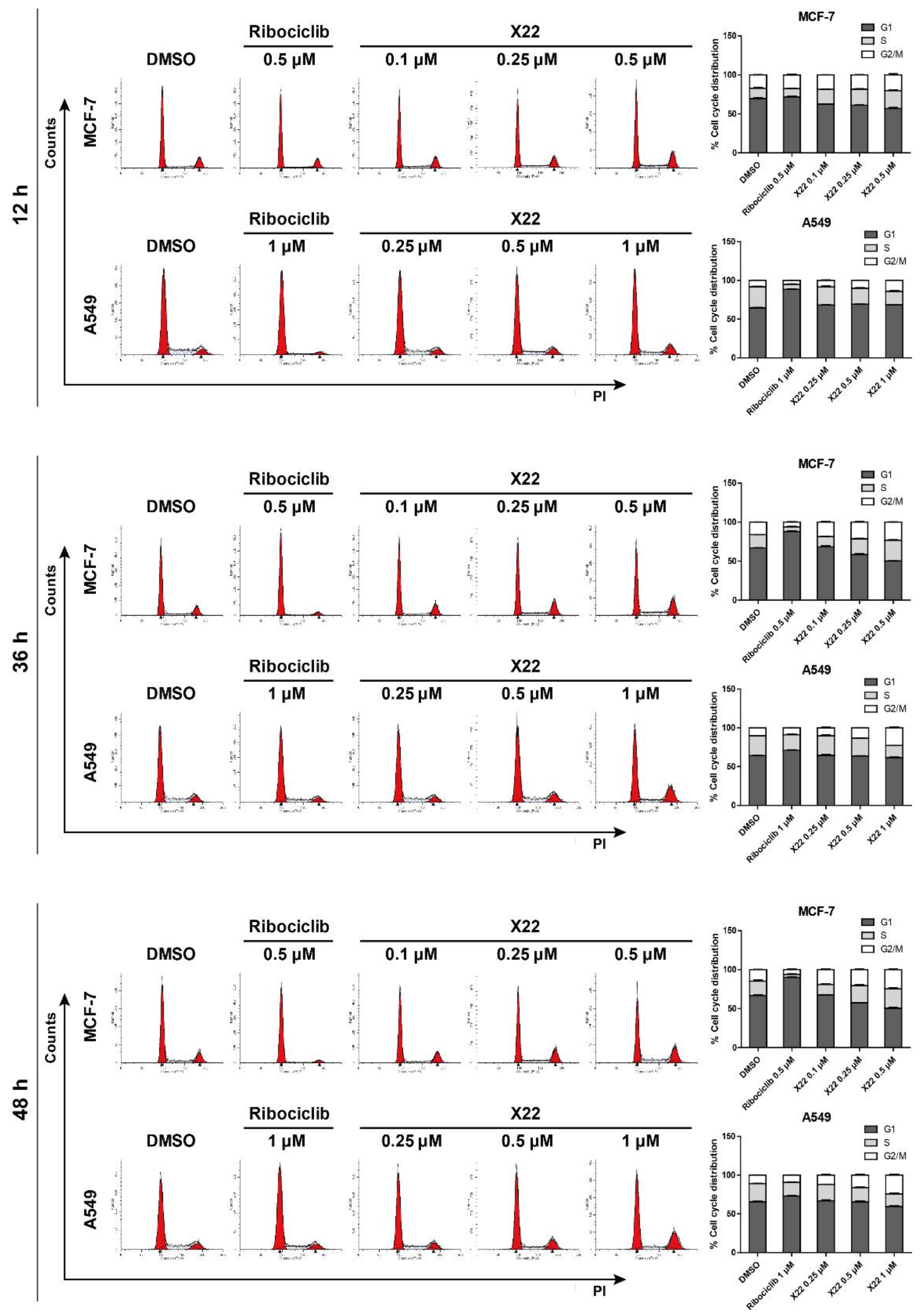

Figure S3. X22 induces G2/M phase arrest in breast cancer and NSCLC cells. The percentages of cell cycle distributions at different time points were analyzed by flow cytometry. Experiments were performed in triplicate.

General Methods for Chemistry.

The commercially obtained chemicals were used directly without further purification. Solvents were purified and distilled following the standard procedures. All the reactions were monitored by thin-layer chromatography (TLC). The NMR spectra were taken on a Bruker AV-400 MHz spectrometer (400 MHz for ^{1}H and 101 MHz for ^{13}C) and chemical shifts were expressed in ppm downfield using tetramethylsilane as the internal standard. High-resolution mass spectra (HRMS) were performed on a VG ZAB-HS mass spectrometer under eluectron spray ionization (ESI). All the derivatives for testing bioactivity were purified to >95% purity which was determined by HPLC analysis on a Shimadzu Prominence-i LC-2030C 3D system (column, InertSustain C18, 4.6 mm × 250 mm, 5 μM; mobile phase, gradient elution of methanol/H_2O (90:10); low rate, 1.0 mL/min; UV wavelength, 190−800 nm; temperapture, 40 °C; injection volume, 10 μL).

1.1. General method A for preparation of **3a**~**3e**

Acetone (reaction concentration: 0.2 mmol/mL) and dibromoalkyl (3.0 eq) were added to a 250 mL round-bottom flask with magnetic stirrer. potassium phthalimide (1.0 eq) was added slowly over 10 min with strong agitation. Then the mixture was heated to 70 °C under reflux overnight. The reaction mixture was filtered and the filtrate was concentrated via rotary evaporation. The residue was purified through silica gel column chromatography to afford the intermediates **3a**~**3e**.

*1.1.1. 2-(3-Bromopropyl)isoindoline-1,3-dione (**3a**).* White solid, Yield 59%; ^{1}H NMR (400 MHz, $CDCl_3$) δ 7.84 (dd, *J* = 5.5, 3.0 Hz, 2H), 7.72 (dd, *J* = 5.5, 3.0 Hz, 2H), 3.83 (t, *J* = 6.8 Hz, 2H), 3.41 (t, *J* = 6.7 Hz, 2H), 2.25 (m, 2H); ^{13}C NMR (101 MHz, $CDCl_3$) δ 168.4, 134.2, 132.1, 123.4, 36.8, 31.7, 29.9.

*1.1.2. 2-(4-Bromobutyl)isoindoline-1,3-dione (**3b**).* White solid, Yield 83%; ^{1}H NMR (400 MHz, $CDCl_3$) δ 7.83 (dd, *J* = 5.4, 3.1 Hz, 2H), 7.71 (dd, *J* = 5.5, 3.0 Hz, 2H), 3.71 (t, *J* = 6.6 Hz, 2H), 3.43 (t, *J* = 6.3 Hz, 2H), 1.92 – 1.82 (m, 4H); ^{13}C NMR (101 MHz, CDCl3) δ 168.5, 134.1, 132.1, 123.4, 37.1, 32.9, 29.9, 27.3.

*1.1.3. 2-(5-Bromopentyl)isoindoline-1,3-dione (**3c**).* White solid, Yield 75%; ^{1}H NMR (400 MHz, $CDCl_3$) δ 7.85 – 7.79 (m, 2H), 7.73 – 7.67 (m, 2H), 3.68 (t, *J* = 7.2 Hz, 2H), 3.38 (t, *J* = 6.8 Hz, 2H), 1.90 (m, 2H), 1.70 (m, 2H), 1.48 (m, 2H); ^{13}C NMR (101 MHz, CDCl3) δ 168.5, 134.0, 132.2, 123.3, 37.7, 33.5, 32.3, 27.8, 25.5.

*1.1.4. 2-(6-Bromohexyl)isoindoline-1,3-dione (**3d**).* White solid, Yield 58%; ^{1}H NMR (400 MHz, $CDCl_3$) δ 7.83 (q, *J* = 4.5 Hz, 2H), 7.75 – 7.66 (m, 2H), 3.68 (t, *J* = 7.2 Hz, 2H), 3.38 (t, *J* = 6.8 Hz, 2H), 1.88 – 1.80 (m, 2H), 1.68 (q, *J* = 7.7 Hz, 2H), 1.47 (q, *J* = 7.5 Hz, 2H), 1.37 (q, *J* = 7.9 Hz, 2H); ^{13}C NMR (101 MHz, CDCl3) δ 168.6, 134.0, 132.3, 123.3, 38.0, 33.8, 32.7, 28.5, 27.8, 26.1.

*1.1.5. 2-(7-Bromoheptyl)isoindoline-1,3-dione (**3e**).* Colorless oil, Yield 80%; ^{1}H NMR (400 MHz, $CDCl_3$) δ 7.83 (dd, *J* = 5.4, 3.1 Hz, 2H), 7.70 was(dd, *J* = 5.5, 3.0 Hz, 2H), 3.70 – 3.64 (m, 2H), 3.38 (t, *J* = 6.8 Hz, 2H), 1.83 (p, *J* = 6.9 Hz, 2H), 1.67 (m, 2H), 1.45 – 1.32 (m, 6H); ^{13}C NMR (101 MHz, $CDCl_3$) δ 168.6, 134.0, 132.2, 123.3, 38.0, 34.0, 32.8, 28.6, 28.4, 28.1, 26.8.

1.2. General method B for preparation of **4a~4e**

The potassium thioacetate (3.0 eq) was added to a stirred solution of intermediates **3a~3e** (13 mmol, 11.0 eq) in 150 mL THF. Then heated to 75 °C under reflux for 5 h, The reaction mixture was filtered and the filtrate was evaporated to brown oil in vacuo. Water was added to the residue purified and stirred vigorously for 30 min. Solid particle was precipitated from the mixture. Filtered and recrystallized to get the intermediates **4a~4e** as white powder.

*1.2.1. S-(3-(1,3-dioxoisoindolin-2-yl)propyl) ethanethioate (**4a**).* Yield 96%; ^{1}H NMR (400 MHz, $CDCl_3$) δ 7.83 (dd, *J* = 5.5, 3.1 Hz, 2H), 7.71 (dd, *J* = 5.4, 3.2 Hz, 2H), 3.74 (t, *J* = 6.9 Hz, 2H), 2.89 (t, *J* = 7.2 Hz, 2H), 2.31 (s, 3H), 1.96 (m, 2H); ^{13}C NMR (101 MHz, $CDCl_3$) δ 195.5, 168.4, 134.1, 132.2, 123.4, 37.0, 30.7, 28.8, 26.5.

*1.2.2. S-(4-(1,3-dioxoisoindolin-2-yl)butyl) ethanethioate (**4b**).* Yield 97%; ^{1}H NMR (400 MHz, $CDCl_3$) δ 7.83 (dd, *J* = 5.5, 3.0 Hz, 2H), 7.70 (dd, *J* = 5.5, 3.1 Hz, 2H), 3.68 (t, *J* = 7.1 Hz, 2H), 2.89 (t, *J* = 7.1 Hz, 2H), 2.30 (s, 3H), 1.77 – 1.70 (m, 2H), 1.66 – 1.58 (m, 2H); ^{13}C NMR (101 MHz, $CDCl_3$) δ 195.8, 168.5, 134.0, 132.2, 123.3, 37.5, 30.7, 28.6, 27.8, 27.0.

*1.2.3. S-(5-(1,3-dioxoisoindolin-2-yl)pentyl) ethanethioate (**4c**).* Yield 99%; ^{1}H NMR (400 MHz, $CDCl_3$) δ 7.82 (dd, *J* = 5.6, 3.0 Hz, 2H), 7.70 (dd, *J* = 5.4, 3.2 Hz, 2H), 3.66 (t, *J* = 7.0 Hz, 2H), 2.85 (t, *J* = 7.2 Hz, 2H), 2.30 (s, 3H), 1.68 (m, 2H), 1.60 – 1.54 (m, 2H), 1.46 – 1.38 (m, 2H); ^{13}C NMR (101 MHz, $CDCl_3$) δ 196.0, 168.4, 134.0, 132.3, 123.5, 37.8, 30.7, 29.4, 28.5, 27.1, 26.5.

*1.2.4. S-(6-(1,3-dioxoisoindolin-2-yl)hexyl) ethanethioate (**4d**)*. Yield 95%; ^{1}H NMR (400 MHz, $CDCl_3$) δ 7.82 (dd, *J* = 5.6, 3.1 Hz, 2H), 7.69 (dd, *J* = 5.4, 3.1 Hz, 2H), 3.65 (t, *J* = 7.2 Hz, 2H), 2.83 (t, *J* = 7.2 Hz, 2H), 2.29 (s, 3H), 1.65 (m, 2H), 1.58 – 1.51 (m, 2H), 1.42 – 1.32 (m, 4H); ^{13}C NMR (101 MHz, $CDCl_3$) δ 196.0, 168.5, 134.0, 132.3, 123.3, 38.0, 30.7, 29.4, 29.1, 28.5, 28.4, 26.5.

*1.2.5. S-(7-(1,3-dioxoisoindolin-2-yl)heptyl) ethanethioate (**4e**)*. Yield 43%; ^{1}H NMR (400 MHz, $CDCl_3$) δ 7.85 – 7.79 (m, 2H), 7.69 (dd, *J* = 5.3, 3.2 Hz, 2H), 3.65 (t, *J* = 7.3 Hz, 2H), 2.83 (t, *J* = 7.4 Hz, 2H), 2.29 (s, 3H), 1.65 (m, 2H), 1.53 (m, 2H), 1.32 (m, 6H); ^{13}C NMR (101 MHz, $CDCl_3$) δ 196.1, 168.5, 134.0, 132.3, 123.3, 38.1, 30.7, 29.5, 29.2, 28.7, 28.7, 28.6, 26.8.

1.3. General method C for preparation of **5a~5e**

Concentrated hydrochloride (9 mL) was added dropwise slowly to a stirred solution of intermediates **4a~4e** (23mmol) in anhydrous methanol under N_2. After that the mixture was heated to 60 °C for 4 h, then cooled to room temperature. The reaction mixture solution was quenched with water and extracted with ethyl acetate (60 mL) for three times. The organic layer was washed with saturated NaCl solution and dried with anhydrous Na_2SO_4. After filtered, the filtrate was concentrated to get crude product in vacuo. The crude product was purified through column chromatography on silica gel to get the intermediates **5a~5e** as light-yellow solid.

*1.3.1. 2-(3-Mercaptopropyl)isoindoline-1,3-dione (**5a**)*. Yield 58%; ^{1}H NMR (400 MHz, DMSO-d_6) δ 7.87 – 7.76 (m, 4H), 3.65 (t, *J* = 6.1 Hz, 2H), 2.48 – 2.44 (m, 2H),

2.41 – 2.27 (m, 1H), 1.85 (m, 2H); ^{13}C NMR (101 MHz, DMSO-d_6) δ 167.9, 134.3, 131.6, 122.8, 36.1, 32.2, 21.3.

*1.3.2. 2-(4-Mercaptobutyl)isoindoline-1,3-dione (**5b**).* Yield 69%; ^{1}H NMR (400 MHz, $CDCl_3$) δ 7.83 (dd, *J* = 5.6, 3.1 Hz, 2H), 7.71 (d, *J* = 4.7 Hz, 2H), 3.69 (t, *J* = 7.0 Hz, 2H), 2.56 (m, 2H), 1.78 (m, 2H), 1.69 – 1.63 (m, 2H), 1.34 (t, *J* = 7.9 Hz, 1H); ^{13}C NMR (101 MHz, $CDCl_3$) δ 168.5, 134.1, 132.2, 123.4, 37.4, 31.2, 27.4, 24.2.

*1.3.3. 2-(5-Mercaptopentyl)isoindoline-1,3-dione (**5c**).* Yield 76%; ^{1}H NMR (400 MHz, $CDCl_3$) δ 7.82 (dd, *J* = 5.3, 3.1 Hz, 2H), 7.69 (dd, *J* = 5.4, 3.1 Hz, 2H), 3.66 (t, *J* = 7.2 Hz, 2H), 2.49 (m, 2H), 1.64 (m, 4H), 1.43 (m, 2H), 1.31 (t, *J* = 7.8 Hz, 1H); ^{13}C NMR (101 MHz, $CDCl_3$) δ 168.5, 134.0, 132.2, 123.3, 37.9, 33.6, 28.1, 25.6, 24.5.

*1.3.4. 2-(6-Mercaptohexyl)isoindoline-1,3-dione (**5d**).* Yield 83%; ^{1}H NMR (400 MHz, $CDCl_3$) δ 7.83 (dd, *J* = 5.4, 3.1 Hz, 2H), 7.70 (dd, *J* = 5.5, 3.1 Hz, 2H), 3.67 (t, *J* = 7.2 Hz, 2H), 2.50 (m, 2H), 1.64 (m, 4H), 1.38 (m, 5H); ^{13}C NMR (101 MHz, $CDCl_3$) δ 168.6, 134.0, 132.3, 123.3, 38.0, 34.0, 28.6, 28.0, 26.4, 24.6.

*1.3.5. 2-(7-Mercaptoheptyl)isoindoline-1,3-dione (**5e**).* Yield 92%; ^{1}H NMR (400 MHz, $CDCl_3$) δ 7.81 (dt, *J* = 9.4, 4.9 Hz, 2H), 7.68 (m, 2H), 3.64 (t, *J* = 7.3, 2H), 2.47 (m, 2H), 1.56 (m, 4H), 1.37 – 1.27 (m, 7H); ^{13}C NMR (101 MHz, $CDCl_3$) δ 168.5, 134.0, 132.2, 123.2, 38.0, 34.0, 28.7, 28.6, 28.3, 26.8, 24.7.

1.4. General method D for preparation of **7a~7h**

1-Fluoro-4-nitrobenzene (1.1 eq) was added slowly to a stirred mixture solution of intermediates **5a~5e** (12 mmol, 1.0 eq) and potassium carbonate (2.0 eq) in 60 mL

DMF. Stirred at room temperature for 4.5 h. Until no more start materials could be detected by TLC, the reaction was quenched with water (50 mL) and continued to stir for 30 min. Solid particle was precipitated from the solution. Filtered via suction ,washed the residue with water and dried to get the intermediates **7a~5h** as yellow solid.

*1.4.1. 2-(3-((4-Nitrophenyl)thio)propyl)isoindoline-1,3-dione (7**a**).* Yield 89%; ^{1}H NMR (400 MHz, $CDCl_3$) δ 8.11 – 8.07 (m, 2H), 7.84 (dt, *J* = 4.9, 2.4 Hz, 2H), 7.73 (dd, *J* = 5.5, 3.0 Hz, 2H), 7.32 – 7.29 (m, 2H), 3.85 (t, *J* = 6.8 Hz, 2H), 3.06 (t, *J* = 7.4 Hz, 2H), 2.11 – 2.07 (m, 2H); ^{13}C NMR (101 MHz, $CDCl_3$) δ 168.4, 147.0, 145.3, 134.3, 132.1, 126.7, 124.1, 123.5, 37.0, 29.6, 27.8.

*1.4.2. 2-(4-((4-Nitrophenyl)thio)butyl)isoindoline-1,3-dione (7**b**).* Yield 38%; ^{1}H NMR (400 MHz, $CDCl_3$) δ 8.10 – 8.04 (m, 2H), 7.83 (dd, *J* = 5.5, 3.0 Hz, 2H), 7.71 (dd, *J* = 5.4, 3.0 Hz, 2H), 7.32 – 7.27 (m, 2H), 3.72 (t, *J* = 6.8 Hz, 2H), 3.06 (t, *J* = 7.1 Hz, 2H), 1.91 – 1.84 (m, 2H), 1.75 (m, 2H); ^{13}C NMR (101 MHz, $CDCl_3$) δ 168.5, 147.4, 145.1, 134.2, 132.1, 126.5, 124.1, 123.4, 37.3, 31.5, 27.7, 25.7.

*1.4.3. 2-(5-((4-Nitrophenyl)thio)pentyl)isoindoline-1,3-dione (7**c**).* Yield 89%; ^{1}H NMR (400 MHz, $CDCl_3$) δ 8.09 (d, *J* = 8.6 Hz, 2H), 7.83 (dd, *J* = 5.4, 3.1 Hz, 2H), 7.71 (dd, *J* = 5.6, 3.1 Hz, 2H), 7.29 (d, *J* = 8.6 Hz, 2H), 3.69 (t, *J* = 7.1 Hz, 2H), 3.00 (t, *J* = 7.4 Hz, 2H), 1.78 – 1.69 (m, 4H), 1.56 – 1.47 (m, 2H); ^{13}C NMR (101 MHz, $CDCl_3$) δ 168.5, 147.9, 145.0, 134.1, 132.2, 126.2, 124.0, 123.3, 37.7, 31.8, 28.8, 28.1, 26.1.

*1.4.4. 2-(6-((4-Nitrophenyl)thio)hexyl)isoindoline-1,3-dione (**7d**).* Yield 90%; ^{1}H NMR (400 MHz, $CDCl_3$) δ 8.11 (d, *J* = 8.6 Hz, 2H), 7.84 (dd, *J* = 5.5, 3.0 Hz, 2H), 7.72 (dd, *J* = 5.4, 3.1 Hz, 2H), 7.30 (d, *J* = 8.7 Hz, 2H), 3.69 (t, *J* = 7.2 Hz, 2H), 3.01 (t, *J* = 7.3 Hz, 2H), 1.73 (d, *J* = 7.3 Hz, 4H), 1.53 (m, 2H), 1.40 (m, 2H); ^{13}C NMR (101 MHz, $CDCl_3$) δ 168.5, 148.0, 145.0, 134.0, 132.2, 126.2, 124.0, 123.3, 37.9, 31.9, 29.1, 28.5, 28.4, 26.4.

*1.4.5. 2-(7-((4-Nitrophenyl)thio)heptyl)isoindoline-1,3-dione (**7e**).* Yield 95%; ^{1}H NMR (400 MHz, $CDCl_3$) δ 8.14 – 8.08 (m, 2H), 7.83 (dd, *J* = 5.5, 3.0 Hz, 2H), 7.71 (dd, *J* = 5.5, 3.1 Hz, 2H), 7.32 – 7.27 (m, 2H), 3.67 (t, *J* = 7.3 Hz, 2H), 2.99 (t, *J* = 7.3 Hz, 2H), 1.73 – 1.65 (m, 4H), 1.46 (m, 2H), 1.37 (m, 4H); ^{13}C NMR (101 MHz, $CDCl_3$) δ 168.6, 148.2, 145.0, 134.0, 132.2, 126.1, 124.1, 123.3, 38.0, 32.0, 28.8, 28.7, 28.6, 28.5, 26.7.

*1.4.6. 2-(5-((4-Nitro-3-(trifluoromethyl)phenyl)thio)pentyl)isoindoline-1,3-dione (**7f**).* Intermediate **7f** was prepared using 4-fluoro-1-nitro-2-(trifluoromethyl)benzene (**6d**) instead of **6a** with the method D in 95% yield; ^{1}H NMR (400 MHz, $CDCl_3$) δ 7.88 (d, *J* = 8.6 Hz, 1H), 7.83 (dd, *J* = 5.5, 3.1 Hz, 2H), 7.72 (dd, *J* = 5.5, 3.0 Hz, 2H), 7.57 (d, *J* = 2.0 Hz, 1H), 7.46 (dd, *J* = 8.6, 2.1 Hz, 1H), 3.70 (t, *J* = 7.1 Hz, 2H), 3.03 (t, *J* = 7.3 Hz, 2H), 1.80 – 1.70 (m, 4H), 1.52 (m, 2H); ^{13}C NMR (101 MHz, $CDCl_3$) δ 168.5, 146.6, 134.1, 132.2, 129.2, 126.1, 125.4, 125.3, 125.3, 124.8, 123.4, 120.6, 37.6, 32.0, 28.1, 27.9, 26.0.

*1.4.7. 2-(5-((3-Methyl-4-nitrophenyl)thio)pentyl)isoindoline-1,3-dione (**7g**).* Intermediate **7g** was prepared using 4-fluoro-2-methyl-1-nitrobenzene (**6b**) instead of

6a with the method D in 94% yield; ^{1}H NMR (400 MHz, $CDCl_3$) δ 7.97 – 7.92 (m, 1H), 7.83 (dd, *J* = 5.4, 3.1 Hz, 2H), 7.71 (dq, *J* = 6.6, 3.9 Hz, 2H), 7.12 (d, *J* = 7.5 Hz, 2H), 3.69 (t, *J* = 7.1 Hz, 2H), 2.98 (t, *J* = 7.3 Hz, 2H), 2.59 (s, 3H), 1.78 – 1.69 (m, 4H), 1.51 (m, 2H); ^{13}C NMR (101 MHz, $CDCl_3$) δ 168.5, 147.1, 145.7, 134.9, 134.1, 132.2, 129.9, 125.6, 124.2, 123.3, 37.7, 31.8, 28.3, 28.2, 26.1, 21.3.

*1.4.8. 2-(5-((3-Methoxy-4-nitrophenyl)thio)pentyl)isoindoline-1,3-dione (**7h**).* Intermediate **7h** was prepared using 4-fluoro-2-methoxy-1-nitrobenzene (**6c**) instead of **6a** with the method D in 79% yield; ^{1}H NMR (400 MHz, $CDCl_3$) δ 7.85 – 7.81 (m, 3H), 7.71 (dd, *J* = 5.7, 3.0 Hz, 2H), 6.86 (d, *J* = 1.8 Hz, 1H), 6.81 (dd, *J* = 8.6, 1.8 Hz, 1H), 3.94 (s, 3H), 3.68 (t, *J* = 7.2 Hz, 2H), 2.98 (t, *J* = 7.3 Hz, 2H), 1.73 (m, 4H), 1.54 – 1.46 (m, 2H); ^{13}C NMR (101 MHz, $CDCl_3$) δ 168.5, 153.6, 147.4, 136.3, 134.1, 132.1, 126.7, 123.3, 117.5, 111.1, 56.7, 37.7, 32.0, 28.2, 28.1, 26.1.

1.5. General method E for preparation of **12a~12e**

Potassium carbonate (2.0 eq) was added slowly to solution of **5a~5e** (9 mmol, 1.0 eq) in 20 mL DMF and the mixture was stirred for 10 min. 1-(bromomethyl)-4-nitrobenzene (1.2 eq) was added to the mixture solution above. Continuous stirring at room temperature for 4.5 h until no more start materials could be detected by TLC, the reaction was quenched with water (30 mL) and extracted with ethyl acetate (30 mL×3). Organic layer was washed with saturated $NaHCO_3$ and NaCl in turn, then dried with anhydrous Na_2SO_4. Through filtered via suction, the crude product was concentrated in vacuo. The pure product was recrystallized within

mixture solution of ethyl acetate and petroleum ether to give the intermediates **12a**~**12e** as light-yellow solid.

*1.5.1. 2-(3-((4-Nitrobenzyl)thio)propyl)isoindoline-1,3-dione (**12a**).* Yield 87%; ^{1}H NMR (400 MHz, $CDCl_3$) δ 8.12 – 8.05 (m, 2H), 7.83 (dd, J = 5.3, 3.1 Hz, 2H), 7.72 (dd, J = 5.5, 3.1 Hz, 2H), 7.44 (d, J = 8.4 Hz, 2H), 3.78 – 3.73 (m, 4H), 2.41 (t, J = 7.4 Hz, 2H), 1.93 (m, 2H); ^{13}C NMR (101 MHz, $CDCl_3$) δ 168.4, 147.0, 146.1, 134.2, 132.1, 129.8, 123.9, 123.4, 37.0, 35.5, 28.5, 27.9.

*1.5.2. 2-(4-((4-Nitrobenzyl)thio)butyl)isoindoline-1,3-dione (**12b**).* Yield 72%; ^{1}H NMR (400 MHz, $CDCl_3$) δ 8.14 (d, J = 8.6 Hz, 2H), 7.83 (dd, J = 5.5, 3.0 Hz, 2H), 7.71 (dd, J = 5.4, 3.1 Hz, 2H), 7.49 – 7.44 (m, 2H), 3.75 (s, 2H), 3.66 (t, J = 7.0 Hz, 2H), 2.45 (t, J = 7.3 Hz, 2H), 1.77 – 1.71 (m, 2H), 1.62 – 1.55 (m, 2H); ^{13}C NMR (101 MHz, $CDCl_3$) δ 168.5, 147.0, 146.5, 134.1, 132.1, 129.7, 123.9, 123.3, 37.4, 35.9, 31.2, 27.8, 26.4.

*1.5.3. 2-(5-((4-Nitrobenzyl)thio)pentyl)isoindoline-1,3-dione (**12c**).* Yield 87%; ^{1}H NMR (400 MHz, $CDCl_3$) δ 8.17 – 8.13 (m, 2H), 7.82 (dd, J = 5.4, 3.1 Hz, 2H), 7.70 (dd, J = 5.4, 3.1 Hz, 2H), 7.48 – 7.44 (m, 2H), 3.74 (s, 2H), 3.65 (t, J = 7.2 Hz, 2H), 2.38 (t, J = 7.3 Hz, 2H), 1.67 – 1.57 (m, 4H), 1.43 – 1.36 (m, 2H); ^{13}C NMR (101 MHz, $CDCl_3$) δ 168.5, 147.0, 146.6, 134.0, 132.2, 129.7, 123.8, 123.3, 37.7, 35.8, 31.3, 28.6, 28.2, 26.0.

*1.5.4. 2-(6-((4-Nitrobenzyl)thio)hexyl)isoindoline-1,3-dione (**12d**).* Yield 90%; ^{1}H NMR (400 MHz, $CDCl_3$) δ 8.19 – 8.13 (m, 2H), 7.83 (dd, J = 5.4, 3.1 Hz, 2H), 7.70 (dd, J = 5.5, 3.1 Hz, 2H), 7.49 – 7.44 (m, 2H), 3.74 (s, 2H), 3.65 (t, J = 7.3 Hz, 2H),

2.40 – 2.36 (m, 2H), 1.67 – 1.62 (m, 2H), 1.58 – 1.51 (m, 2H), 1.41 – 1.30 (m, 4H); ^{13}C NMR (101 MHz, $CDCl_3$) δ 168.6, 147.0, 146.6, 134.0, 132.2, 129.7, 123.9, 123.3, 37.9, 35.8, 31.5, 29.0, 28.5, 28.4, 26.5.

*1.5.5. 2-(7-((4-Nitrobenzyl)thio)heptyl)isoindoline-1,3-dione (**12e**)*. Yield 76%; ^{1}H NMR (400 MHz, $CDCl_3$) δ 8.17 (dd, *J* = 8.7, 3.6 Hz, 2H), 7.84 (p, *J* = 4.7, 3.8 Hz, 2H), 7.71 (p, *J* = 4.3 Hz, 2H), 7.47 (dd, *J* = 8.6, 3.7 Hz, 2H), 3.75 (s, 2H), 3.66 (t, *J* = 7.4 Hz, 2H), 2.38 (t, *J* = 7.5 Hz, 2H), 1.66 (m, 2H), 1.54 (m, 2H), 1.37 – 1.28 (m, 6H); ^{13}C NMR (101 MHz, $CDCl_3$) δ 168.6, 150.0, 146.7, 134.0, 132.3, 129.7, 123.9, 123.3, 38.0, 35.9, 31.6, 29.0, 28.8, 28.7, 28.6, 26.7.

1.6. General method F for preparation of **8a~8e** and **13a~13e**

A solution of intermediates **7a**~**7b** or **12a**~**12e** (2.5 mmol) in dichloromethane (20 mL) were cooled to 0 °C in ice-water bath. A solution of 85% m-CPBA (4.5 eq) of DCM (10 mL) was added dropwise over 10 min keeping the temperature at 0 °C. When the addition was finished, the mixture was warm to room temperature and stirred for 2 h. After completion of reaction, the reaction was quenched with saturated Na_2SO_3 solution and stirred for a while. The organic phase was washed with brine, dried with anhydrous Na_2SO_4. After filtered, the product was concentrated with rotary evaporator to give the intermediates **8a~8e** or **13a~13e** as white solid.

*1.6.1. 2-(3-((4-Nitrophenyl)sulfonyl)propyl)isoindoline-1,3-dione (**8a**)*. Yield 98%; ^{1}H NMR (400 MHz, $CDCl_3$) δ 8.39 (d, *J* = 8.4 Hz, 2H), 8.11 (d, *J* = 8.3 Hz, 2H), 7.87 – 7.80 (m, 2H), 7.74 (t, *J* = 4.3 Hz, 2H), 3.80 (t, *J* = 6.8 Hz, 2H), 3.24 (t, *J* = 7.7 Hz,

2H), 2.15 (m, 2H); ^{13}C NMR (101 MHz, $CDCl_3$) δ 168.3, 144.5, 134.5, 131.9, 129.9, 127.4, 124.7, 123.6, 54.0, 36.3, 22.4.

1.6.2. 2-(4-((4-Nitrophenyl)sulfonyl)butyl)isoindoline-1,3-dione ***(8b)***. Yield 90%; ^{1}H NMR (400 MHz, $CDCl_3$) δ 8.10 – 8.05 (m, 2H), 7.83 (dd, *J* = 5.4, 3.0 Hz, 2H), 7.71 (dd, *J* = 5.3, 3.0 Hz, 2H), 7.29 (d, *J* = 8.9 Hz, 2H), 3.71 (t, *J* = 6.8 Hz, 2H), 3.06 (t, *J* = 7.1 Hz, 2H), 1.91 – 1.83 (m, 2H), 1.76 (m, 2H); ^{13}C NMR (101 MHz, $CDCl_3$) δ 168.5, 147.4, 134.2, 134.1, 132.0, 126.4, 124.1, 123.4, 37.3, 31.4, 27.7, 25.7.

1.6.3. 2-(5-((4-Nitrophenyl)sulfonyl)pentyl)isoindoline-1,3-dione ***(8c)***. Yield 99%; ^{1}H NMR (400 MHz, $CDCl_3$) δ 8.40 (d, *J* = 8.5 Hz, 2H), 8.10 (d, *J* = 8.4 Hz, 2H), 7.81 (dt, *J* = 8.2, 3.8 Hz, 2H), 7.71 (dd, *J* = 5.6, 3.1 Hz, 2H), 3.63 (t, *J* = 7.0 Hz, 2H), 3.16 – 3.11 (m, 2H), 1.77 (m, 2H), 1.69 – 1.63 (m, 2H), 1.44 (m, 2H); ^{13}C NMR (101 MHz, $CDCl_3$) δ 168.5, 151.0, 144.8, 134.2, 132.1, 129.7, 124.6, 123.4, 56.0, 37.4, 28.1, 25.5, 22.2.

1.6.4. 2-(6-((4-Nitrophenyl)sulfonyl)hexyl)isoindoline-1,3-dione ***(8d)***. Yield 99%; ^{1}H NMR (400 MHz, $CDCl_3$) δ 8.42 (d, *J* = 8.5 Hz, 2H), 8.11 (d, *J* = 8.5 Hz, 2H), 7.86 – 7.78 (m, 2H), 7.72 (dd, *J* = 5.7, 3.0 Hz, 2H), 3.64 (t, *J* = 7.1 Hz, 2H), 3.13 (t, *J* = 8.2 Hz, 2H), 1.67 (m, 4H), 1.43 (m, 2H), 1.32 (m, 2H); ^{13}C NMR (101 MHz, $CDCl_3$) δ 168.5, 151.0, 144.9, 134.1, 132.2, 129.8, 124.7, 123.4, 56.2, 37.7, 28.2, 27.8, 26.2, 22.6.

1.6.5. 2-(7-((4-Nitrophenyl)sulfonyl)heptyl)isoindoline-1,3-dione ***(8e)***. Yield 98%; ^{1}H NMR (400 MHz, $CDCl_3$) δ 8.47 – 8.39 (m, 2H), 8.16 – 8.09 (m, 2H), 7.83 (dq, *J* = 7.2, 4.3, 3.7 Hz, 2H), 7.71 (dt, *J* = 5.4, 3.4 Hz, 2H), 3.64 (t, *J* = 7.3 Hz, 2H), 3.16 –

3.09 (m, 2H), 1.72 – 1.61 (m, 4H), 1.31 (m, 6H); ^{13}C NMR (101 MHz, $CDCl_3$) δ 168.6, 151.0, 144.9, 134.1, 132.2, 129.7, 124.6, 123.3, 56.2, 37.8, 28.5, 28.4, 28.2, 26.4, 22.6.

*1.6.6. 2-(3-((4-Nitrobenzyl)sulfonyl)propyl)isoindoline-1,3-dione (**13a**)*. Yield 98%; ^{1}H NMR (400 MHz, $CDCl_3$) δ 8.19 (d, *J* = 8.3 Hz, 2H), 7.85 (dt, *J* = 7.6, 3.7 Hz, 2H), 7.76 (dd, *J* = 5.5, 3.1 Hz, 2H), 7.58 (d, *J* = 8.3 Hz, 2H), 4.32 (s, 2H), 3.81 (t, *J* = 6.5 Hz, 2H), 3.00 – 2.94 (m, 2H), 2.23 (m, 2H); ^{13}C NMR (101 MHz, $CDCl_3$) δ 168.3, 164.6, 134.6, 134.6, 131.9, 131.8, 124.3, 123.6, 58.8, 49.8, 36.3, 21.8.

*1.6.7. 2-(4-((4-Nitrobenzyl)sulfonyl)butyl)isoindoline-1,3-dione (**13b**)*. Yield 99%; ^{1}H NMR (400 MHz, $CDCl_3$) δ 8.28 – 8.22 (m, 2H), 7.84 (dt, *J* = 7.1, 3.5 Hz, 2H), 7.73 (dd, *J* = 5.4, 3.1 Hz, 2H), 7.61 (d, *J* = 8.7 Hz, 2H), 4.32 (s, 2H), 3.71 (t, *J* = 6.3 Hz, 2H), 3.02 (m, 2H), 1.91 – 1.81 (m, 4H); ^{13}C NMR (101 MHz, $CDCl_3$) δ 168.5, 148.5, 134.8, 134.3, 132.0, 131.9, 124.2, 123.5, 58.8, 51.7, 36.8, 27.4, 19.3.

*1.6.8. 2-(5-((4-Nitrobenzyl)sulfonyl)pentyl)isoindoline-1,3-dione (**13c**)*. Yield 96%; ^{1}H NMR (400 MHz, $CDCl_3$) δ 8.28 – 8.24 (m, 2H), 7.84 (dd, *J* = 5.4, 3.1 Hz, 2H), 7.73 (dd, *J* = 5.4, 3.1 Hz, 2H), 7.65 – 7.61 (m, 2H), 4.32 (s, 2H), 3.69 (t, *J* = 7.0 Hz, 2H), 2.92 – 2.87 (m, 2H), 1.95 – 1.88 (m, 2H), 1.75 – 1.68 (m, 2H), 1.50 – 1.43 (m, 2H); ^{13}C NMR (101 MHz, $CDCl_3$) δ 168.6, 148.5, 135.0, 134.2, 132.1, 131.9, 124.3, 123.4, 58.6, 52.0, 37.3, 28.1, 25.6, 21.5.

*1.6.9. 2-(6-((4-Nitrobenzyl)sulfonyl)hexyl)isoindoline-1,3-dione (**13d**)*. Yield 93%; ^{1}H NMR (400 MHz, $CDCl_3$) δ 8.31 – 8.23 (m, 2H), 7.83 (dd, *J* = 5.5, 3.1 Hz, 2H), 7.72 (dd, *J* = 5.5, 3.0 Hz, 2H), 7.61 (d, *J* = 8.6 Hz, 2H), 4.31 (s, 2H), 3.67 (t, *J* = 7.1

Hz, 2H), 2.93 – 2.87 (m, 2H), 1.85 (m, 2H), 1.71 – 1.65 (m, 2H), 1.51 – 1.44 (m, 2H), 1.36 (m, 2H); ^{13}C NMR (101 MHz, $CDCl_3$) δ 168.6, 148.4, 135.0, 134.1, 132.2, 131.8, 124.3, 123.4, 58.6, 52.1, 37.6, 28.2, 27.9, 26.2, 21.9.

*1.6.10. 2-(7-((4-Nitrobenzyl)sulfonyl)heptyl)isoindoline-1,3-dione (**13e**)*. Yield 95%; ^{1}H NMR (400 MHz, $CDCl_3$) δ 8.30 – 8.25 (m, 2H), 7.83 (dd, *J* = 5.5, 3.0 Hz, 2H), 7.71 (dd, *J* = 5.5, 3.0 Hz, 2H), 7.61 (d, *J* = 8.5 Hz, 2H), 4.31 (s, 2H), 3.66 (t, *J* = 7.2 Hz, 2H), 2.93 – 2.85 (m, 2H), 1.83 (m, 2H), 1.66 (m, 2H), 1.42 – 1.32 (m, 6H); ^{13}C NMR (101 MHz, $CDCl_3$) δ 168.6, 148.5, 135.0, 134.1, 132.2, 131.8, 124.3, 123.3, 58.7, 52.2, 37.8, 28.5, 28.4, 28.3, 26.4, 21.8.

1.7. General method E1 and E2 for preparation of **9a~10e** and **14a~15e**

Method E1: To a stirred solution of the yellow solid **7a**, **7c**, **7f~7h**, **8a~8e** or **13a~13e** (1.0 eq) in AcOH (reaction concentration: 0.1 mmol/mL), Fe powder (11 eq) was added in portion, and the mixture was refluxed at 65 °C. After the completion of the reaction, the reaction mixture was absorbed onto celite, washed with dichloromethane, concentrated under reduced pressure. The crude product was purified by silica gel column chromatography to afford intermediates **9a~10e** or **15a~15e** as yellow oil.

Method E2: To a stirred solution of the yellow solid **12a~13e** (1.0 eq) and ammonium chloride (9.0 eq) in MeOH (reaction concentration: 0.1 mmol/mL), Zn powder (7.0 eq) was added in several portion, and the mixture was stirred at RT for 1.5h. After the completion of the reaction, the reaction mixture was absorbed onto

celite, concentrated under reduced pressure. The crude product was purified by silica gel column chromatography to afford intermediates **14a~14e** as yellow oil.

*1.7.1. 2-(3-((4-Aminophenyl)thio)propyl)isoindoline-1,3-dione **(9a)***. Yield 70%; ^{1}H NMR (400 MHz, $CDCl_3$) δ 7.83 (ddt, J = 7.4, 5.0, 2.5 Hz, 2H), 7.73 – 7.68 (m, 2H), 7.29 – 7.24 (m, 2H), 6.63 – 6.57 (m, 2H), 3.77 (m, 4H), 2.78 (t, J = 7.2 Hz, 2H), 1.92 (m, 2H); ^{13}C NMR (101 MHz, $CDCl_3$) δ 168.4, 146.3, 134.8, 134.0, 132.2, 123.3, 122.6, 115.7, 37.1, 34.1, 28.3.

*1.7.2. 2-(5-((4-Aminophenyl)thio)pentyl)isoindoline-1,3-dione **(9b)***. Yield 75%; ^{1}H NMR (400 MHz, DMSO-d_6) δ 7.87 – 7.80 (m, 4H), 7.07 – 7.01 (m, 2H), 6.53 – 6.47 (m, 2H), 5.19 (s, 2H), 3.53 (t, *J* = 7.0 Hz, 2H), 2.66 (t, *J* = 7.1 Hz, 2H), 1.55 (m, 2H), 1.50 – 1.43 (m, 2H), 1.34 (m, 2H); ^{13}C NMR (101 MHz, DMSO-d_6) δ 167.9, 148.2, 134.3, 133.5, 131.6, 123.0, 119.0, 114.4, 37.3, 35.4, 28.3, 27.5, 25.1.

*1.7.3. 2-(5-((4-Amino-3-(trifluoromethyl)phenyl)thio)pentyl)isoindoline-1,3-dione **(9c)***. Yield 95%; ^{1}H NMR (400 MHz, DMSO-d_6) δ 7.83 (h, *J* = 4.4 Hz, 4H), 7.30 (d, *J* = 8.7 Hz, 2H), 6.79 (d, *J* = 8.2 Hz, 1H), 5.74 (d, *J* = 6.0 Hz, 2H), 3.53 (t, *J* = 6.9 Hz, 2H), 2.73 (td, *J* = 7.2, 2.2 Hz, 2H), 1.59 – 1.54 (m, 2H), 1.47 (m, 2H), 1.35 (m, 2H); ^{13}C NMR (101 MHz, DMSO-d_6) δ 167.9, 145.6, 136.9, 134.3, 131.6, 129.6, 129.6, 129.5, 129.5, 126.0, 122.9, 119.4, 117.7, 110.8, 37.2, 35.0, 28.2, 27.4, 25.1.

*1.7.4. 2-(5-((4-Amino-3-methylphenyl)thio)pentyl)isoindoline-1,3-dione **(9d)***. Yield 52%; ^{1}H NMR (400 MHz, DMSO-d_6) δ 7.86 – 7.80 (m, 4H), 6.96 (d, *J* = 2.1 Hz, 1H), 6.92 (dd, *J* = 8.2, 2.2 Hz, 1H), 6.53 (d, *J* = 8.1 Hz, 1H), 4.95 (s, 2H), 3.53 (t, *J* = 7.0 Hz, 2H), 2.66 (t, *J* = 7.1 Hz, 2H), 2.00 (s, 3H), 1.59 – 1.52 (m, 2H), 1.50 – 1.43 (m,

2H), 1.34 (m, 2H); ^{13}C NMR (101 MHz, DMSO-d_6) δ 167.9, 146.2, 134.3, 134.1, 131.6, 130.9, 123.0, 121.8, 119.3, 114.4, 37.3, 35.4, 28.3, 27.5, 25.1, 17.3.

1.7.5. 2-(5-((4-Amino-3-methoxyphenyl)thio)pentyl)isoindoline-1,3-dione ***(9e)***. Yield 47%; ^{1}H NMR (400 MHz, DMSO-d_6) δ 7.83 (ddd, *J* = 9.9, 5.2, 3.2 Hz, 4H), 6.79 (d, *J* = 1.9 Hz, 1H), 6.72 (dd, *J* = 8.0, 1.9 Hz, 1H), 6.56 (d, *J* = 8.0 Hz, 1H), 4.82 (s, 2H), 3.74 (s, 3H), 3.53 (t, *J* = 7.0 Hz, 2H), 2.72 (t, *J* = 7.1 Hz, 2H), 1.60 – 1.53 (m, 2H), 1.52 – 1.45 (m, 2H), 1.36 (m, 2H); ^{13}C NMR (101 MHz, DMSO-d_6) δ 167.9, 146.3, 137.3, 134.3, 131.6, 125.2, 123.0, 119.8, 114.4, 113.8, 55.3, 37.3, 35.2, 28.3, 27.5, 25.2.

1.7.6. 2-(3-((4-Aminophenyl)sulfonyl)propyl)isoindoline-1,3-dione ***(10a)***. Yield 88%; ^{1}H NMR (400 MHz, $CDCl_3$) δ 7.82 (dt, *J* = 7.8, 3.9 Hz, 2H), 7.72 (dd, *J* = 5.6, 3.1 Hz, 2H), 7.63 (d, *J* = 8.4 Hz, 2H), 6.68 (d, *J* = 8.3 Hz, 2H), 4.25 (s, 2H), 3.75 (t, *J* = 6.8 Hz, 2H), 3.15 – 3.06 (m, 2H), 2.07 (m, 2H); ^{13}C NMR (101 MHz, $CDCl_3$) δ 168.3, 158.9, 151.6, 134.3, 132.0, 130.4, 123.5, 114.3, 54.5, 36.5, 22.9.

1.7.7. 2-(4-((4-Aminophenyl)sulfonyl)butyl)isoindoline-1,3-dione ***(10b)***. Yield 71%; ^{1}H NMR (400 MHz, $CDCl_3$) δ 7.81 (dd, *J* = 5.5, 3.1 Hz, 2H), 7.70 (dd, *J* = 5.5, 3.1 Hz, 2H), 7.65 – 7.58 (m, 2H), 6.70 – 6.65 (m, 2H), 4.23 (s, 2H), 3.67 – 3.62 (m, 2H), 3.11 – 3.05 (m, 2H), 1.78 – 1.71 (m, 4H); ^{13}C NMR (101 MHz, $CDCl_3$) δ 168.4, 151.6, 134.1, 132.1, 130.3, 127.0, 123.4, 114.2, 56.0, 37.1, 27.3, 20.5.

1.7.8. 2-(5-((4-Aminophenyl)sulfonyl)pentyl)isoindoline-1,3-dione ***(10c)***. Yield 23%; ^{1}H NMR (400 MHz, $CDCl_3$) δ 7.81 (dt, *J* = 7.6, 3.7 Hz, 2H), 7.70 (dd, *J* = 5.5, 3.1 Hz, 2H), 7.61 (d, *J* = 8.3 Hz, 2H), 6.68 (d, *J* = 8.3 Hz, 2H), 4.24 (s, 2H), 3.63 (t, *J*

= 7.1 Hz, 2H), 3.04 – 2.97 (m, 2H), 1.71 (m, 2H), 1.64 (m, 2H), 1.39 (m, 2H); ^{13}C NMR (101 MHz, $CDCl_3$) δ 168.5, 151.5, 134.1, 132.2, 130.2, 127.1, 123.3, 114.2, 56.5, 37.7, 28.3, 25.7, 22.7.

*1.7.9. 2-(6-((4-Aminophenyl)sulfonyl)hexyl)isoindoline-1,3-dione (**10d**)*. Yield 75%; ^{1}H NMR (400 MHz, $CDCl_3$) δ 7.82 (dd, *J* = 5.4, 3.0 Hz, 2H), 7.70 (dd, *J* = 5.5, 3.0 Hz, 2H), 7.63 – 7.58 (m, 2H), 6.71 – 6.67 (m, 2H), 4.25 (s, 2H), 3.63 (t, *J* = 7.1 Hz, 2H), 3.03 – 2.97 (m, 2H), 1.69 – 1.59 (m, 4H), 1.41 – 1.33 (m, 2H), 1.28 (m, 2H); ^{13}C NMR (101 MHz, $CDCl_3$) δ 168.5, 151.5, 134.1, 132.2, 130.2, 127.0, 123.3, 114.2, 56.6, 37.8, 28.4, 28.0, 26.4, 23.0.

*1.7.10. 2-(7-((4-Aminophenyl)sulfonyl)heptyl)isoindoline-1,3-dione (**10e**)*. Yield 41%; ^{1}H NMR (400 MHz, $CDCl_3$) δ 7.91 – 7.79 (m, 2H), 7.76 – 7.55 (m, 4H), 6.72 (m, 2H), 4.18 (s, 2H), 3.65 (t, *J* =8.6 Hz, 2H), 3.09 – 2.94 (m, 2H), 1.71 – 1.62 (m, 4H), 1.31 (m, 6H); ^{13}C NMR (101 MHz, $CDCl_3$) δ 168.5, 151.4, 134.0, 132.3, 130.3, 127.6, 123.3, 114.3, 56.8, 38.0, 28.7, 28.5, 28.3, 26.6, 23.0.

*1.7.11. 2-(3-((4-Aminobenzyl)thio)propyl)isoindoline-1,3-dione (**14a**)*. Yield 32%; ^{1}H NMR (400 MHz, DMSO-d_6) δ 7.84 (qd, *J* = 5.3, 4.2, 2.1 Hz, 4H), 6.90 – 6.87 (m, 2H), 6.45 – 6.40 (m, 2H), 4.97 (s, 2H), 3.62 (t, *J* = 6.8 Hz, 2H), 3.54 (s, 2H), 2.37 (t, *J* = 7.3 Hz, 2H), 1.85 – 1.80 (m, 2H); ^{13}C NMR (101 MHz, DMSO-d_6) δ 168.0, 147.4, 134.3, 131.7, 129.4, 124.7, 123.0, 113.7, 36.7, 34.6, 27.6, 27.6.

*1.7.12. 2-(4-((4-Aminobenzyl)thio)butyl)isoindoline-1,3-dione (**14b**)*. Yield 61%; ^{1}H NMR (400 MHz, $CDCl_3$) δ 7.85 – 7.70 (m, 4H), 7.06 – 6.99 (m, 2H), 6.60 – 6.48 (m, 2H), 3.66 (t, *J* = 7.3 Hz, 2H), 3.60 (s, 2H), 2.38 (t, *J* = 7.4 Hz, 2H), 1.68 – 1.58 (m,

2H), 1.50 – 1.41 (m, 2H); ^{13}C NMR (101 MHz, $CDCl_3$) δ 168.6, 146.0, 134.1, 132.0, 129.8, 128.6, 123.3, 116.4, 38.2, 35.9, 31.2, 27.8, 26.4.

1.7.13. 2-(5-((4-Aminobenzyl)thio)pentyl)isoindoline-1,3-dione ***(14c)***. Yield 48%; ^{1}H NMR (400 MHz, $CDCl_3$) δ 7.83 (dt, *J* = 5.7, 2.9 Hz, 2H), 7.70 (dd, *J* = 5.5, 3.0 Hz, 2H), 7.12 – 7.03 (m, 2H), 6.67 – 6.59 (m, 2H), 3.66 (t, *J* = 7.3 Hz, 2H), 3.60 (s, 2H), 2.40 – 2.35 (m, 2H), 1.67 – 1.56 (m, 4H), 1.42 – 1.37 (m, 2H); ^{13}C NMR (101 MHz, $CDCl_3$) δ 168.6, 145.0, 134.0, 132.2, 129.9, 128.6, 123.3, 115.4, 38.0, 35.9, 31.0, 28.9, 28.3, 26.2.

1.7.14. 2-(6-((4-Aminobenzyl)thio)hexyl)isoindoline-1,3-dione ***(14d)***. Yield 49%; ^{1}H NMR (400 MHz, $CDCl_3$) δ 7.83 (dd, *J* = 5.4, 3.0 Hz, 2H), 7.70 (dd, *J* = 5.5, 3.0 Hz, 2H), 7.08 (d, *J* = 7.9 Hz, 2H), 6.69 (d, *J* = 7.9 Hz, 2H), 3.66 (t, *J* = 7.3 Hz, 2H), 3.60 (s, 2H), 2.37 (t, *J* = 7.3 Hz, 2H), 1.66 – 1.61 (m, 2H), 1.56 – 1.49 (m, 2H), 1.34 (m, 4H); ^{13}C NMR (101 MHz, $CDCl_3$) δ 168.6, 134.0, 132.3, 129.9, 129.6, 124.7, 123.3, 116.2, 38.1, 35.9, 31.2, 29.2, 28.6, 28.5, 26.6.

1.7.15. 2-(7-((4-Aminobenzyl)thio)heptyl)isoindoline-1,3-dione ***(14e)***. Yield 63%; ^{1}H NMR (400 MHz, DMSO-d_6) δ 7.85 (m, 4H), 6.99 – 6.88 (m, 2H), 6.49 (m, 2H), 5.00 (s, 2H), 3.55 (m, 4H), 2.32 (t, *J* = 6.8 Hz, 2H), 1.57 (m, 2H), 1.46 (m, 2H), 1.25 (m, 6H); ^{13}C NMR (101 MHz, DMSO-d_6) δ 168.0, 147.4, 134.4, 131.6, 129.4, 125.0, 123.0, 113.7, 37.3, 34.9, 30.4, 28.7, 28.6, 28.1, 27.9, 26.1.

1.7.16. 2-(3-((4-Aminobenzyl)sulfonyl)propyl)isoindoline-1,3-dione ***(15a)***. Yield 58%; ^{1}H NMR (400 MHz, $CDCl_3$) δ 7.85 (td, *J* = 5.5, 2.9 Hz, 2H), 7.73 (td, *J* = 5.2, 2.7 Hz, 2H), 7.19 – 7.09 (m, 2H), 6.66 – 6.56 (m, 2H), 4.14 – 4.07 (m, 2H), 3.96 –

3.54 (m, 4H), 2.92 – 2.84 (m, 2H), 2.19 – 2.10 (m, 2H); ^{13}C NMR (101 MHz, $CDCl_3$) δ 168.2, 147.4, 134.3, 132.1, 131.6, 123.5, 117.1, 115.4, 59.3, 48.4, 36.5, 21.8.

*1.7.17. 2-(4-((4-Aminobenzyl)sulfonyl)butyl)isoindoline-1,3-dione (**15b**).* Yield 58%; ^{1}H NMR (400 MHz, $CDCl_3$) δ 7.83 (dd, *J* = 5.4, 3.1 Hz, 2H), 7.72 (dd, *J* = 5.5, 3.0 Hz, 2H), 7.17 – 7.12 (m, 2H), 6.67 – 6.63 (m, 2H), 4.10 (s, 2H), 3.81 (s, 2H), 3.68 (t, *J* = 6.4 Hz, 2H), 2.86 (dd, *J* = 9.0, 5.9 Hz, 2H), 1.79 (dtt, *J* = 8.7, 6.7, 4.3 Hz, 4H); ^{13}C NMR (101 MHz, $CDCl_3$) δ 168.4, 147.4, 134.2, 132.1, 131.7, 123.4, 117.2, 115.5, 59.3, 50.1, 37.1, 27.5, 19.3.

*1.7.18. 2-(5-((4-Aminobenzyl)sulfonyl)pentyl)isoindoline-1,3-dione (**15c**).* Yield 42%; ^{1}H NMR (400 MHz, $CDCl_3$) δ 7.83 (dd, *J* = 5.4, 3.1 Hz, 2H), 7.71 (dd, *J* = 5.4, 3.0 Hz, 2H), 7.15 (d, *J* = 8.5 Hz, 2H), 6.69 – 6.64 (m, 2H), 4.09 (s, 2H), 3.66 (t, *J* = 7.1 Hz, 4H), 2.80 – 2.75 (m, 2H), 1.85 – 1.77 (m, 2H), 1.66 (d, *J* = 7.8 Hz, 2H), 1.44 – 1.37 (m, 2H); ^{13}C NMR (101 MHz, $CDCl_3$) δ 168.5, 147.4, 134.1, 132.2, 131.6, 123.4, 117.3, 115.5, 59.2, 50.4, 37.5, 28.2, 25.9, 21.5.

*1.7.19. 2-(6-((4-Aminobenzyl)sulfonyl)hexyl)isoindoline-1,3-dione (**15d**).* Yield 50%; ^{1}H NMR (400 MHz, $CDCl_3$) δ 7.86 – 7.81 (m, 2H), 7.71 (dd, *J* = 5.5, 3.1 Hz, 2H), 7.17 – 7.13 (m, 2H), 6.69 – 6.65 (m, 2H), 4.09 (s, 2H), 3.81 (s, 2H), 3.65 (t, *J* = 7.2 Hz, 2H), 2.80 – 2.74 (m, 2H), 1.80 – 1.73 (m, 2H), 1.68 – 1.63 (m, 2H), 1.44 – 1.31 (m, 4H); ^{13}C NMR (101 MHz, $CDCl_3$) δ 168.5, 147.4, 134.1, 132.2, 131.6, 123.3, 117.4, 115.5, 59.2, 50.5, 37.8, 28.3, 28.1, 26.4, 21.8.

*1.7.20. 2-(7-((4-Aminobenzyl)sulfonyl)heptyl)isoindoline-1,3-dione (**15e**).* Yield 76%; ^{1}H NMR (400 MHz, $CDCl_3$) δ 7.84 (dd, *J* = 5.4, 3.1 Hz, 2H), 7.71 (dt, *J* = 5.4,

2.7 Hz, 2H), 7.18 – 7.14 (m, 2H), 6.71 – 6.66 (m, 2H), 4.09 (s, 2H), 3.82 (s, 2H), 3.65 (d, *J* = 7.2 Hz, 2H), 2.79 – 2.74 (m, 2H), 1.77 (d, *J* = 4.7 Hz, 2H), 1.68 – 1.64 (m, 2H), 1.38 – 1.31 (m, 6H); ^{13}C NMR (101 MHz, $CDCl_3$) δ 168.6, 147.4, 134.0, 132.3, 131.6, 123.3, 117.6, 115.5, 59.3, 50.7, 38.0, 28.6, 28.6, 28.4, 26.6, 21.9.

1.8. General method F for preparation of **17a~20e**

The intermediates **9a~10e** or **14a~15e** (1.0 eq), 2-chloro-7-cyclopentyl-*N*,*N*-dimethyl-7H-pyrrolo[2,3-*d*]pyrimidine-6-carboxamide (**16**, 1.0 eq), $Pd(OAc)_2$ (0.1 eq), BINAP (0.06 eq), Cs_2CO_3 (2.0 eq) were dissolved in 1,4-dioxane and degassed with argon. The resulted mixture was heated to 105 °C for 7 h. After monitored by TLC to observe completion of reaction, the reaction mixture was filtered through Celite after cooling to 25 °C, then the solvents were removed in vacuo. The crude product was purified by silica gel column chromatography to afford intermediates **17a~20e** as light-yellow solid.

*1.8.1. 7-Cyclopentyl-2-((4-((3-(1,3-dioxoisoindolin-2-yl)propyl)thio)phenyl)amino)-N,N-dimethyl-7H-pyrrolo[2,3-d]pyrimidine-6-carboxamide (**17a**).* Yield 41%; ^{1}H NMR (400 MHz, $CDCl_3$) δ 8.64 (s, 1H), 7.82 (d, *J* = 7.2, 2H), 7.72 – 7.67 (m, 2H), 7.67 – 7.63 (m, 2H), 7.40 (m, 3H), 6.42 (s, 1H), 4.77 (m, 1H), 3.82 (t, *J* = 6.7 Hz, 2H), 3.15 (s, 6H), 2.88 (t, *J* = 7.2 Hz, 2H), 2.58 (m, 2H), 2.04 (m, 4H), 2.00 – 1.96 (m, 2H), 1.68 (m, 2H); ^{13}C NMR (101 MHz, $CDCl_3$) δ 168.4, 164.2, 155.3, 152.1, 151.7, 139.7, 134.1, 133.1, 132.2, 132.2, 127.0, 123.4, 119.1, 112.6, 101.1, 58.1, 39.6, 37.1, 33.5, 30.3, 28.4, 24.8.

*1.8.2. 7-Cyclopentyl-2-((4-((5-(1,3-dioxoisoindolin-2-yl)pentyl)thio)phenyl)amino)-N,N-dimethyl-7H-pyrrolo[2,3-d]pyrimidine-6-carboxamide (**17b**).* Yield 49%; ^{1}H NMR (400 MHz, $CDCl_3$) δ 8.63 (s, 1H), 7.82 (dd, *J* = 5.4, 3.0 Hz, 2H), 7.69 (dd, *J* = 5.4, 3.0 Hz, 2H), 7.66 – 7.62 (m, 2H), 7.46 (s, 1H), 7.36 – 7.32 (m, 2H), 6.42 (s, 1H), 4.80 – 4.72 (m, 1H), 3.67 (t, *J* = 7.2 Hz, 2H), 3.15 (s, 6H), 2.85 (t, *J* = 7.3 Hz, 2H), 2.57 (m, 2H), 2.09 – 2.00 (m, 4H), 1.67 (m, 6H), 1.50 – 1.44 (m, 2H); ^{13}C NMR (101 MHz, $CDCl_3$) δ 168.5, 164.2, 155.3, 152.1, 151.5, 139.1, 134.0, 132.2, 132.2, 132.0, 128.0, 123.3, 119.2, 112.5, 101.1, 58.1, 38.0, 35.4, 30.3, 29.8, 29.1, 28.3, 26.1, 24.8.

*1.8.3. 7-Cyclopentyl-2-((4-((5-(1,3-dioxoisoindolin-2-yl)pentyl)thio)-2-(trifluoromethyl)phenyl)amino)-N,N-dimethyl-7H-pyrrolo[2,3-d]pyrimidine-6-carboxamide (**17c**).* Yield 27%; ^{1}H NMR (400 MHz, $CDCl_3$) δ 8.66 (s, 1H), 8.44 (d, *J* = 8.7 Hz, 1H), 7.83 (dd, *J* = 5.5, 3.1 Hz, 2H), 7.70 (dd, *J* = 5.4, 3.0 Hz, 2H), 7.59 (d, *J* = 2.2 Hz, 1H), 7.50 (dd, *J* = 8.7, 2.2 Hz, 1H), 7.46 (s, 1H), 6.44 (s, 1H), 4.76 (m, 1H), 3.68 (t, *J* = 7.2 Hz, 2H), 3.14 (s, 6H), 2.89 (t, *J* = 7.3 Hz, 2H), 2.49 – 2.41 (m, 2H), 2.08 – 2.02 (m, 2H), 1.97 – 1.89 (m, 2H), 1.67 (m, 6H), 1.52 – 1.45 (m, 2H); ^{13}C NMR (101 MHz, $CDCl_3$) δ 168.5, 164.0, 155.0, 151.9, 151.6, 136.5, 134.4, 134.0, 132.7, 132.2, 129.4, 128.4, 128.3, 125.5, 123.3, 122.9, 113.3, 100.9, 100.1, 57.9, 39.5, 37.9, 34.9, 30.6, 28.9, 28.3, 26.1, 25.0.

1.8.4. 7-Cyclopentyl-2-((4-((5-(1,3-dioxoisoindolin-2-yl)pentyl)thio)-2-methylphenyl)amino)

-N,N-dimethyl-7H-pyrrolo[2,3-d]pyrimidine-6-carboxamide ***(17d)***. Yield 33%; ^{1}H NMR (400 MHz, $CDCl_3$) δ 8.62 (s, 1H), 8.21 (d, *J* = 8.2 Hz, 1H), 7.82 (dd, *J* = 5.5, 3.0 Hz, 2H), 7.69 (dd, *J* = 5.5, 3.1 Hz, 2H), 7.24 (m, 2H), 6.92 (s, 1H), 6.40 (s, 1H), 4.74 (m, 1H), 3.67 (t, *J* = 7.2 Hz, 2H), 3.14 (s, 6H), 2.84 (d, *J* = 7.4 Hz, 2H), 2.53 – 2.46 (m, 2H), 2.31 (s, 3H), 2.05 – 1.94 (m, 4H), 1.66 (m, 6H), 1.51 – 1.45 (m, 2H); ^{13}C NMR (101 MHz, $CDCl_3$) δ 168.5, 164.2, 155.8, 152.1, 151.8, 137.2, 134.0, 133.0, 132.2, 132.0, 129.2, 128.7, 127.9, 123.3, 120.6, 112.5, 101.0, 57.9, 38.0, 35.2, 30.5, 29.8, 29.1, 28.3, 26.1, 24.9, 18.1.

1.8.5. 7-Cyclopentyl-2-((4-((5-(1,3-dioxoisoindolin-2-yl)pentyl)thio)-2-methoxyphenyl)amino)-N,N-dimethyl-7H-pyrrolo[2,3-d]pyrimidine-6-carboxamide ***(17e)***. Yield 24%; ^{1}H NMR (400 MHz, $CDCl_3$) δ 8.64 (s, 1H), 8.55 (d, *J* = 8.4 Hz, 1H), 7.81 (m, 3H), 7.69 (dd, *J* = 5.5, 3.1 Hz, 2H), 7.03 (dd, *J* = 8.4, 1.9 Hz, 1H), 6.94 (d, *J* = 1.9 Hz, 1H), 6.42 (s, 1H), 4.77 (m, 1H), 3.91 (s, 3H), 3.67 (t, *J* = 7.2 Hz, 2H), 3.14 (s, 6H), 2.86 (t, *J* = 7.3 Hz, 2H), 2.62 (m, 2H), 2.06 (m, 4H), 1.72 – 1.65 (m, 6H), 1.50 – 1.44 (m, 2H); ^{13}C NMR (101 MHz, $CDCl_3$) δ 168.5, 164.2, 155.3, 152.1, 151.7, 147.8, 134.0, 132.2, 131.9, 129.1, 127.0, 124.0, 123.3, 117.9, 113.4, 112.3, 101.1, 58.1, 56.0, 38.0, 35.7, 30.2, 29.8, 29.1, 28.3, 26.1, 24.7.

1.8.6. 7-Cyclopentyl-2-((4-((3-(1,3-dioxoisoindolin-2-yl)propyl)sulfonyl)phenyl)amino)-N,N-dimethyl-7H-pyrrolo[2,3-d]pyrimidine-6-carboxamide ***(18a)***. Yield 44%; ^{1}H NMR (400 MHz, DMSO-d_6) δ 10.14 (s, 1H), 8.83 (s, 1H), 8.08 – 8.04 (m, 2H), 7.83 – 7.74

(m, 6H), 6.65 (s, 1H), 4.81 – 4.73 (m, 1H), 3.65 (t, *J* = 6.9 Hz, 2H), 3.39 – 3.34 (m, 2H), 2.47 – 2.39 (m, 2H), 2.04 – 1.96 (m, 4H), 1.92 – 1.87 (m, 2H), 1.65 (m, 2H); ^{13}C NMR (101 MHz, DMSO-d_6) δ 167.9, 162.8, 154.6, 152.0, 150.8, 145.9, 134.2, 132.6, 131.8, 129.2, 128.8, 122.9, 117.3, 112.5, 100.5, 62.8, 57.0, 52.8, 36.0, 29.7, 24.2, 21.9.

1.8.7. 7-Cyclopentyl-2-((4-((4-(1,3-dioxoisoindolin-2-yl)butyl)sulfonyl)phenyl)amino)-N,N-dimethyl-7H-pyrrolo[2,3-d]pyrimidine-6-carboxamide ***(18b)***. Yield 54%; ^{1}H NMR (400 MHz, $CDCl_3$) δ 8.70 (s, 1H), 7.97 (s, 1H), 7.90 (d, *J* = 8.9 Hz, 2H), 7.82 (d, *J* = 7.2 Hz, 2H), 7.79 (dd, *J* = 5.5, 3.0 Hz, 2H), 7.65 (dd, *J* = 5.6, 3.1 Hz, 2H), 6.48 (s, 1H), 4.80 (m, 1H), 3.65 (m, 2H), 3.16 (m, 8H), 2.60 – 2.53 (m, 2H), 2.08 (m, 4H), 1.80 – 1.73 (m, 6H); ^{13}C NMR (101 MHz, $CDCl_3$) δ 168.3, 163.9, 154.4, 151.7, 151.5, 145.3, 134.1, 133.1, 132.1, 130.3, 129.5, 123.4, 117.7, 113.4, 100.9, 58.2, 56.0, 37.0, 30.3, 29.8, 27.2, 24.8, 20.4.

1.8.8. 7-Cyclopentyl-2-((4-((5-(1,3-dioxoisoindolin-2-yl)pentyl)sulfonyl)phenyl)amino)-N,N-dimethyl-7H-pyrrolo[2,3-d]pyrimidine-6-carboxamide ***(18c)***. Yield 69%; ^{1}H NMR (400 MHz, $CDCl_3$) δ 8.69 (s, 1H), 7.92 – 7.89 (m, 2H), 7.86 (s, 1H), 7.82 (m, 2H), 7.80 (m, 2H), 7.68 (m, 2H), 6.47 (s, 1H), 4.79 (m, 1H), 3.64 (t, *J* = 7.1 Hz, 2H), 3.16 (s, 6H), 3.10 – 3.05 (m, 2H), 2.57 (m, 2H), 2.12 – 2.03 (m, 4H), 1.81 – 1.71 (m, 4H), 1.64 (m, 2H), 1.45 – 1.39 (m, 2H); ^{13}C NMR (101 MHz, $CDCl_3$) δ 168.5, 163.9, 154.4,

151.7, 151.6, 145.3, 134.1, 133.1, 132.1, 130.4, 129.5, 123.3, 117.7, 113.4, 100.9, 58.2, 56.5, 39.5, 37.6, 30.3, 28.3, 25.8, 24.8, 22.7.

*1.8.9. 7-Cyclopentyl-2-((4-((6-(1,3-dioxoisoindolin-2-yl)hexyl)sulfonyl)phenyl)amino)-N,N-dimethyl-7H-pyrrolo[2,3-d]pyrimidine-6-carboxamide (**18d**)*. Yield 54%; ^{1}H NMR (400 MHz, $CDCl_3$) δ 8.70 (s, 1H), 7.91 (d, *J* = 8.8 Hz, 2H), 7.85 (s, 1H), 7.83 – 7.80 (m, 4H), 7.69 (dd, *J* = 5.5, 3.0 Hz, 2H), 6.47 (s, 1H), 4.79 (m, 1H), 3.63 (t, *J* = 7.1 Hz, 2H), 3.16 (s, 6H), 3.10 – 3.05 (m, 2H), 2.61 – 2.53 (m, 2H), 2.12 – 2.04 (m, 4H), 1.72 (dt, *J* = 8.1, 5.4 Hz, 4H), 1.66 – 1.61 (m, 2H), 1.43 – 1.38 (m, 2H), 1.31 (m, 2H); ^{13}C NMR (101 MHz, $CDCl_3$) δ 168.5, 163.9, 154.4, 151.7, 151.6, 145.3, 134.0, 133.1, 132.2, 130.5, 129.5, 123.3, 117.7, 113.4, 100.9, 58.2, 56.6, 39.5, 37.8, 30.3, 28.4, 28.0, 26.4, 24.8, 22.9.

*1.8.10. 7-Cyclopentyl-2-((4-((7-(1,3-dioxoisoindolin-2-yl)heptyl)sulfonyl)phenyl)amino)-N,N-dimethyl-7H-pyrrolo[2,3-d]pyrimidine-6-carboxamide (**18e**)*. Yield 41%; ^{1}H NMR (400 MHz, $CDCl_3$) δ 8.70 (s, 1H), 7.91 (d, *J* = 8.7 Hz, 2H), 7.81 (m, 5H), 7.69 (dd, *J* = 5.5, 3.1 Hz, 2H), 6.47 (s, 1H), 4.79 (m, 1H), 3.63 (t, *J* = 7.2 Hz, 2H), 3.16 (s, 6H), 3.09 – 3.05 (m, 2H), 2.62 – 2.54 (m, 2H), 2.07 (m, 4H), 1.76 – 1.69 (m, 4H), 1.62 (m, 2H), 1.35 – 1.28 (m, 6H); ^{13}C NMR (101 MHz, $CDCl_3$) δ 168.6, 163.9, 154.5, 151.7, 151.7, 145.2, 134.0, 133.0, 132.2, 130.6, 129.5, 123.3, 117.7, 113.5, 100.9, 58.2, 56.7, 39.6, 37.9, 30.3, 28.7, 28.5, 28.3, 26.6, 24.8, 22.9.

1.8.11. 7-Cyclopentyl-2-((4-(((3-(1,3-dioxoisoindolin-2-yl)propyl)thio)methyl)phenyl)amino)-N,N-dimethyl-7H-pyrrolo[2,3-d]pyrimidine-6-carboxamide ***(19a)***. Yield 30%; ^{1}H NMR (400 MHz, $CDCl_3$) δ 8.65 (s, 1H), 7.84 (dd, *J* = 5.3, 3.2 Hz, 2H), 7.69 (dd, *J* = 5.4, 3.1 Hz, 2H), 7.61 (d, *J* = 8.2 Hz, 2H), 7.42 (s, 1H), 7.29 (m, 2H), 6.45 (s, 1H), 4.83 – 4.75 (m, 1H), 3.78 (t, *J* = 7.0 Hz, 2H), 3.74 (s, 2H), 3.18 (s, 6H), 2.60 (m, 2H), 2.48 (t, *J* = 7.4 Hz, 2H), 2.07 (s, 4H), 2.00 – 1.94 (m, 2H), 1.71 (m, 2H); ^{13}C NMR (101 MHz, $CDCl_3$) δ 168.4, 164.2, 155.4, 152.2, 151.6, 139.1, 134.0, 132.2, 132.1, 131.3, 129.4, 123.3, 118.7, 112.4, 101.1, 60.5, 58.1, 37.3, 35.6, 30.3, 28.3, 28.2, 24.8.

1.8.12. 7-Cyclopentyl-2-((4-(((4-(1,3-dioxoisoindolin-2-yl)butyl)thio)methyl)phenyl)amino)-N, N-dimethyl-7H-pyrrolo[2,3-d]pyrimidine-6-carboxamide ***(19b)***. Yield 35%; ^{1}H NMR (400 MHz, $CDCl_3$) δ 8.62 (s, 1H), 7.79 (m, 3H), 7.67 – 7.61 (m, 4H), 7.23 (d, *J* = 8.0 Hz, 2H), 6.40 (s, 1H), 4.78 – 4.71 (m, 1H), 3.66 (m, 4H), 3.12 (s, 6H), 2.57 (m, 2H), 2.45 (t, *J* = 7.2 Hz, 2H), 2.04 (m, 4H), 1.75 – 1.58 (m, 6H); ^{13}C NMR (101 MHz, $CDCl_3$) δ 168.4, 164.2, 155.5, 152.1, 151.7, 139.2, 133.9, 132.1, 131.8, 131.5, 129.2, 123.2, 118.7, 112.3, 101.0, 57.9, 39.5, 37.5, 35.9, 30.8, 30.2, 27.8, 26.5, 24.7.

1.8.13. 7-Cyclopentyl-2-((4-(((5-(1,3-dioxoisoindolin-2-yl)pentyl)thio)methyl)phenyl)amino)-N,N-dimethyl-7H-pyrrolo[2,3-d]pyrimidine-6-carboxamide ***(19c)***. Yield 30%; ^{1}H NMR (400 MHz, $CDCl_3$) δ 8.61 (s, 1H), 7.81 (m, 2H), 7.69 – 7.62 (m, 4H), 7.54 (s, 1H), 7.24-7.25 (m, 2H), 6.41 (s, 1H), 4.75 (m, 1H), 3.69 – 3.63 (m, 4H), 3.14 (s, 6H),

2.57 (m, 2H), 2.41 (t, *J* = 7.5 Hz, 2H), 2.03 (m, 4H), 1.69 – 1.59 (m, 6H), 1.41 (m, 2H); ^{13}C NMR (101 MHz, $CDCl_3$) δ 168.5, 164.2, 155.5, 152.2, 151.6, 139.1, 134.0, 132.2, 132.0, 131.8, 129.3, 123.3, 118.8, 112.3, 101.1, 58.0, 37.9, 35.9, 31.1, 30.3, 29.8, 28.9, 28.3, 26.2, 24.8.

1.8.14. 7-Cyclopentyl-2-((4-(((6-(1,3-dioxoisoindolin-2-yl)hexyl)thio)methyl)phenyl)amino)-N,N-dimethyl-7H-pyrrolo[2,3-d]pyrimidine-6-carboxamide ***(19d)***. Yield 50%; ^{1}H NMR (400 MHz, $CDCl_3$) δ 8.63 (s, 1H), 7.83 (dd, *J* = 5.4, 3.0 Hz, 2H), 7.70 (dd, *J* = 5.5, 3.0 Hz, 2H), 7.67 – 7.64 (m, 2H), 7.43 (s, 1H), 7.27 (d, *J* = 6.7 Hz, 2H), 6.42 (s, 1H), 4.81 – 4.74 (m, 1H), 3.69 (s, 2H), 3.67 (d, *J* = 7.2 Hz, 2H), 3.16 (s, 6H), 2.57 (m, 2H), 2.43 (t, *J* = 7.3 Hz, 2H), 2.08 – 2.02 (m, 4H), 1.67 (m, 4H), 1.57 (m, 2H), 1.41 – 1.31 (m, 4H); ^{13}C NMR (101 MHz, $CDCl_3$) δ 168.6, 164.2, 155.5, 152.2, 151.6, 139.1, 134.0, 132.3, 132.0, 131.9, 129.4, 123.3, 118.7, 112.4, 101.1, 72.9, 58.1, 53.6, 38.1, 35.9, 31.2, 30.3, 29.2, 28.5, 26.6, 24.8.

1.8.15. 7-cyclopentyl-2-((4-(((7-(1,3-dioxoisoindolin-2-yl)heptyl)thio)methyl)phenyl)amino)-N,N-dimethyl-7H-pyrrolo[2,3-d]pyrimidine-6-carboxamide ***(19e)***. Yield 46%; ^{1}H NMR (400 MHz, $CDCl_3$) δ 8.47 (s, 1H), 7.68 (dd, *J* = 5.4, 3.0 Hz, 2H), 7.56 – 7.54 (m, 2H), 7.52 – 7.49 (m, 2H), 7.14 – 7.09 (m, 3H), 6.27 (s, 1H), 4.65 – 4.59 (m, 1H), 3.54 (s, 2H), 3.51 (t, *J* = 7.3 Hz, 2H), 3.00 (s, 6H), 2.44 (m, 2H), 2.26 (t, *J* = 7.4 Hz, 2H), 1.90 (m, 4H), 1.56 – 1.48 (m, 4H), 1.40 (m, 2H), 1.17 (m, 6H); ^{13}C NMR (101 MHz, $CDCl_3$) δ 168.6, 164.2, 155.5, 152.2, 151.6, 139.0, 134.0, 132.3, 132.1, 131.9, 129.4,

123.3, 118.8, 112.4, 101.1, 58.1, 38.1, 36.0, 35.3, 31.3, 30.3, 29.3, 28.9, 28.8, 28.7, 26.8, 24.8.

*1.8.16. 7-cyclopentyl-2-((4-(((3-(1,3-dioxoisoindolin-2-yl)propyl)sulfonyl)methyl)phenyl)amino)-N,N-dimethyl-7H-pyrrolo[2,3-d]pyrimidine-6-carboxamide (**20a**).* Yield 25%; ^{1}H NMR (400 MHz, $CDCl_3$) δ 8.66 (s, 1H), 7.79 (dd, *J* = 5.5, 3.0 Hz, 2H), 7.69 (s, 1H), 7.67 – 7.64 (m, 3H), 7.61 (s, 1H), 7.31 (d, *J* = 8.1 Hz, 2H), 6.44 (s, 1H), 4.77 (m, 1H), 4.20 (s, 2H), 3.76 (t, *J* = 6.6 Hz, 2H), 3.15 (s, 6H), 2.93 – 2.89 (m, 2H), 2.55 (m, 2H), 2.19 – 2.14 (m, 2H), 2.08 – 2.01 (m, 4H), 1.68 (m, 2H); ^{13}C NMR (101 MHz, $CDCl_3$) δ 168.2, 164.1, 155.1, 152.0, 151.7, 141.1, 134.2, 132.3, 131.9, 131.0, 123.5, 120.4, 118.8, 112.7, 101.0, 59.3, 58.0, 48.6, 39.6, 36.4, 30.3, 24.8, 21.8.

*1.8.17. 7-Cyclopentyl-2-((4-(((4-(1,3-dioxoisoindolin-2-yl)butyl)sulfonyl)methyl)phenyl)amino)-N,N-dimethyl-7H-pyrrolo[2,3-d]pyrimidine-6-carboxamide (**20b**).* Yield 28%; ^{1}H NMR (400 MHz, $CDCl_3$) δ 8.65 (s, 1H), 7.83 (dd, *J* = 5.5, 3.0 Hz, 2H), 7.76 – 7.69 (m, 4H), 7.48 (s, 1H), 7.37 – 7.34 (m, 2H), 6.44 (s, 1H), 4.78 (m, 1H), 4.21 (s, 2H), 3.69 (t, *J* = 6.5 Hz, 2H), 3.15 (s, 6H), 2.94 – 2.89 (m, 2H), 2.56 (m, 2H), 2.09 – 2.01 (m, 4H), 1.84 (m, 4H), 1.74 – 1.66 (m, 2H); ^{13}C NMR (101 MHz, $CDCl_3$) δ 168.4, 164.1, 155.0, 152.1, 151.5, 141.1, 134.2, 132.4, 132.1, 131.2, 123.5, 120.7, 118.9, 112.8, 101.1, 59.4, 58.1, 50.3, 39.6, 37.1, 30.3, 27.5, 24.8, 19.3.

1.8.18. 7-Cyclopentyl-2-((4-(((5-(1,3-dioxoisoindolin-2-yl)pentyl)sulfonyl)methyl)phenyl)ami

*no)-N,N-dimethyl-7H-pyrrolo[2,3-d]pyrimidine-6-carboxamide (**20c**)*. Yield 48%; ^{1}H NMR (400 MHz, $CDCl_3$) δ 8.64 (s, 1H), 7.81 (dt, *J* = 8.4, 3.9 Hz, 2H), 7.75 (d, *J* = 8.2 Hz, 2H), 7.69 (dd, *J* = 5.4, 3.1 Hz, 2H), 7.55 (s, 1H), 7.35 (d, *J* = 8.2 Hz, 2H), 6.43 (s, 1H), 4.78 (m, 1H), 4.20 (s, 2H), 3.66 (t, *J* = 7.1 Hz, 2H), 3.15 (s, 6H), 2.83 (t, *J* = 8.0 Hz, 2H), 2.60 – 2.51 (m, 2H), 2.03 (m, 4H), 1.85 (m, 2H), 1.68 (m, 4H), 1.46 – 1.40 (m, 2H); ^{13}C NMR (101 MHz, $CDCl_3$) δ 168.5, 164.1, 155.2, 152.0, 151.7, 141.1, 134.1, 132.3, 132.1, 131.1, 123.4, 120.7, 118.8, 112.8, 101.0, 59.3, 58.0, 50.7, 39.5, 37.5, 30.3, 28.2, 25.8, 24.8, 21.5.

*1.8.19. 7-Cyclopentyl-2-((4-(((6-(1,3-dioxoisoindolin-2-yl)hexyl)sulfonyl)methyl)phenyl)amino)-N,N-dimethyl-7H-pyrrolo[2,3-d]pyrimidine-6-carboxamide (**20d**)*. Yield 58%; ^{1}H NMR (400 MHz, $CDCl_3$) δ 8.65 (s, 1H), 7.82 (dd, *J* = 5.5, 3.1 Hz, 2H), 7.75 (d, *J* = 8.1 Hz, 2H), 7.69 (dd, *J* = 5.5, 3.0 Hz, 2H), 7.52 (s, 1H), 7.35 (d, *J* = 8.1 Hz, 2H), 6.43 (s, 1H), 4.77 (m, 1H), 4.19 (s, 2H), 3.65 (t, *J* = 7.2 Hz, 2H), 3.15 (s, 6H), 2.82 (t, *J* = 6.5 Hz, 2H), 2.56 (m, 2H), 2.04 (m, 4H), 1.80 (m, 2H), 1.68 (m, 4H), 1.42 (m, 2H), 1.34 (m, 2H); ^{13}C NMR (101 MHz, $CDCl_3$) δ 168.5, 164.1, 155.2, 152.0, 151.7, 141.1, 134.1, 132.3, 132.2, 131.1, 123.3, 120.8, 118.9, 112.8, 101.0, 59.2, 58.1, 50.8, 39.5, 37.7, 30.3, 28.3, 28.1, 26.4, 24.8, 21.8.

*1.8.20. 7-Cyclopentyl-2-((4-(((7-(1,3-dioxoisoindolin-2-yl)heptyl)sulfonyl)methyl)phenyl)amino)-N,N-dimethyl-7H-pyrrolo[2,3-d]pyrimidine-6-carboxamide (**20e**)*. Yield 48%; ^{1}H NMR (400 MHz, $CDCl_3$) δ 8.64 (s, 1H), 7.83 (dd, *J* = 5.4, 3.1 Hz, 2H), 7.78 – 7.75 (m,

2H), 7.70 (dd, *J* = 5.4, 3.0 Hz, 2H), 7.54 (s, 1H), 7.36 (d, *J* = 8.2 Hz, 2H), 6.43 (s, 1H), 4.79 (m, 1H), 4.19 (s, 2H), 3.65 (t, *J* = 7.2 Hz, 2H), 3.15 (s, 6H), 2.84 – 2.79 (m, 2H), 2.55 (m, 2H), 2.10 – 2.02 (m, 4H), 1.78 (m, 2H), 1.68 (m, 4H), 1.34 (m, 6H); ^{13}C NMR (101 MHz, $CDCl_3$) δ 168.6, 164.1, 157.0, 154.1, 152.2, 141.1, 134.0, 132.6, 132.3, 131.1, 123.3, 121.0, 119.0, 112.8, 101.1, 59.3, 58.1, 51.0, 38.0, 30.4, 29.8, 28.7, 28.5, 28.4, 26.5, 24.8, 21.9.

1.9. General method G1-G3 for preparation of **X1~X23**

Method G1: To a stirred solution of **17a~20e** (1.0 eq) in MeOH, hydrazine hydrate (80%, 6.0 eq) was added, then stirred 3 h at 60 °C. After removing solvent in vacuo, the residue was dissolved in n-butanol (10 mL) and washed with saturated Na_2CO_3 solution and brine. The organic layer was concentrated under reduced pressure to use directly without purification. After dissolving the resulting light-yellow oil in THF (5 mL), carbon disulphide (20 equiv), DCC (1.4 eq) were added in successively, stirred overnight at RT. The solvent was concentrated in vacuo and crude product was purified by silica gel column chromatography to give target compound **X1~X23** (except **X5~X7**) as light-yellow solid or oil.

Method G2: A solution of compounds **X1** or **X4** (1.0 eq) in dichloromethane (5 mL) were cooled to -10 °C. A solution of 85% m-CPBA (1.5 eq) of DCM (5 mL) was added dropwise over 10 min keeping the temperature below 0 °C. When the addition was finished, the mixture was warm to 0 °C and stirred for 2 h. After completion of reaction, the reaction was quenched with saturated Na_2SO_3 solution and stirred for a while. The organic phase was washed with brine, dried with anhydrous Na_2SO_4. The

crude product was concentrated with rotary evaporator and purified through silica gel column chromatography to give the target compounds **X5** or **X7** as white solid.

Method G3: A solution of compounds **X4** (1.0 eq) in dichloromethane (5 mL) were cooled to 0 °C. A solution of 85% m-CPBA (4.5 eq) of DCM (5 mL) was added dropwise over 10 min keeping the temperature at 0 °C. Then stirred at room temperature for 2 h. The reaction was quenched with saturated Na_2SO_3 solution and stirred for a while. The organic phase was washed with brine, dried with anhydrous Na_2SO_4. The crude product was concentrated with rotary evaporator and purified through silica gel column chromatography to give the target compounds **X6** as white solid.

*1.9.1. 7-Cyclopentyl-2-((4-((5-isothiocyanatopentyl)thio)phenyl)amino)-N,N-dimethyl-7H-pyrrolo[2,3-d]pyrimidine-6-carboxamide (**X1**).* Yield 80%; ^{1}H NMR (400 MHz, $CDCl_3$) δ 8.64 (s, 1H), 7.68 – 7.65 (m, 2H), 7.56 (s, 1H), 7.38 – 7.35 (m, 2H), 6.43 (s, 1H), 4.77 (m, 1H), 3.49 (t, J = 6.5 Hz, 2H), 3.15 (s, 6H), 2.86 (t, J = 7.1 Hz, 2H), 2.62 – 2.55 (m, 2H), 2.09 – 2.02 (m, 4H), 1.69 (m, 6H), 1.58 – 1.53 (m, 2H); ^{13}C NMR (101 MHz, $CDCl_3$) δ 164.1, 155.3, 152.1, 151.7, 139.4, 132.3, 132.3, 132.2, 127.5, 119.2, 112.6, 101.1, 58.0, 45.0, 35.4, 34.1, 30.3, 29.7, 28.6, 25.7, 24.8; HRMS (ESI, m/z) calcd for $C_{26}H_{33}N_6OS_2^+$ $[M + H]^+$, 509.2152, found 509.2154. HPLC purity 96%.

1.9.2. 7-Cyclopentyl-2-((4-((5-isothiocyanatopentyl)thio)-2-methylphenyl)amino)-N,N-dimet

hyl-7H-pyrrolo[2,3-d]pyrimidine-6-carboxamide ***(X2)***. Yield 38%; ^{1}H NMR (400 MHz, $CDCl_3$) δ 8.64 (s, 1H), 8.25 (d, *J* = 8.2 Hz, 1H), 7.29 – 7.26 (m, 2H), 6.99 (s, 1H), 6.42 (s, 1H), 4.76 (m, 1H), 3.51 (t, *J* = 6.5 Hz, 2H), 3.15 (s, 6H), 2.88 (t, *J* = 7.1 Hz, 2H), 2.55 – 2.48 (m, 2H), 2.34 (s, 3H), 2.08 – 1.96 (m, 4H), 1.73 – 1.64 (m, 6H), 1.59 – 1.54 (m, 2H); ^{13}C NMR (101 MHz, $CDCl_3$) δ 164.2, 155.7, 152.2, 151.6, 137.4, 135.2, 133.3, 132.2, 129.5, 128.3, 128.0, 120.6, 112.5, 101.0, 57.9, 45.0, 39.6, 35.2, 30.5, 29.7, 28.6, 25.8, 24.9, 18.2; HRMS (ESI, m/z) calcd for $C_{27}H_{35}N_6OS_2^+$ $[M + H]^+$, 523.2308, found 523.2309. HPLC purity 96%.

1.9.3. 7-Cyclopentyl-2-((4-((5-isothiocyanatopentyl)thio)-2-methoxyphenyl)amino)-N,N-dimethyl-7H-pyrrolo[2,3-d]pyrimidine-6-carboxamide ***(X3)***. Yield 30%; ^{1}H NMR (400 MHz, $CDCl_3$) δ 8.63 (s, 1H), 8.54 (d, *J* = 8.4 Hz, 1H), 7.98 (s, 1H), 7.05 (dd, *J* = 8.4, 2.0 Hz, 1H), 6.96 (d, *J* = 1.9 Hz, 1H), 6.44 (s, 1H), 4.78 (m, 1H), 3.93 (s, 3H), 3.51 (t, *J* = 6.5 Hz, 2H), 3.15 (s, 6H), 2.89 (t, *J* = 7.1 Hz, 2H), 2.66 – 2.56 (m, 2H), 2.06 (q, *J* = 8.6, 8.0 Hz, 4H), 1.75 – 1.65 (m, 6H), 1.60 – 1.54 (m, 2H); ^{13}C NMR (101 MHz, $CDCl_3$) δ 164.1, 155.0, 152.1, 151.4, 147.9, 132.2, 129.2, 126.8, 125.9, 124.2, 118.0, 113.5, 112.4, 101.1, 58.1, 56.0, 45.0, 39.6, 35.6, 30.2, 29.7, 28.7, 25.7, 24.7; HRMS (ESI, m/z) calcd for $C_{27}H_{35}N_6O_2S_2^+$ $[M + H]^+$, 539.2257, found 539.2259. HPLC purity 99%.

1.9.4. 7-Cyclopentyl-2-((4-((5-isothiocyanatopentyl)thio)-2-(trifluoromethyl)phenyl)amino)-N,N-dimethyl-7H-pyrrolo[2,3-d]pyrimidine-6-carboxamide ***(X4)***. Yield 36%; ^{1}H

NMR (400 MHz, $CDCl_3$) δ 8.66 (s, 1H), 8.48 (d, *J* = 8.7 Hz, 1H), 7.61 (d, *J* = 2.2 Hz, 1H), 7.52 (dd, *J* = 8.8, 2.2 Hz, 1H), 7.45 (s, 1H), 6.44 (s, 1H), 4.81 – 4.72 (m, 1H), 3.51 (t, *J* = 6.5 Hz, 2H), 3.14 (s, 6H), 2.91 (t, *J* = 7.1 Hz, 2H), 2.49 – 2.42 (m, 2H), 2.09 – 2.02 (m, 2H), 1.98 – 1.91 (m, 2H), 1.74 – 1.64 (m, 6H), 1.56 (m, 2H); ^{13}C NMR (101 MHz, $CDCl_3$) δ 164.0, 154.9, 151.8, 151.6, 136.8, 134.7, 132.7, 128.8, 128.6, 128.6, 125.5, 122.8, 119.6, 119.3, 113.3, 100.9, 57.9, 45.0, 39.5, 34.9, 30.6, 29.6, 28.5, 25.7, 25.0; HRMS (ESI, m/z) calcd for $C_{27}H_{32}F_3N_6OS_2^+$ $[M + H]^+$, 577.2026, found 577.2024. HPLC purity 98%.

*1.9.5. 7-Cyclopentyl-2-((4-((5-isothiocyanatopentyl)sulfinyl)-2-(trifluoromethyl)phenyl)amino)-N,N-dimethyl-7H-pyrrolo[2,3-d]pyrimidine-6-carboxamide (**X5**).* Yield 35%; ^1H NMR (400 MHz, $CDCl_3$) δ 8.90 (d, *J* = 8.8 Hz, 1H), 8.70 (s, 1H), 7.86 (s, 1H), 7.75 (dd, *J* = 8.8, 2.1 Hz, 1H), 7.67 (s, 1H), 6.46 (s, 1H), 4.79 (m, 1H), 3.52 (t, *J* = 6.4 Hz, 2H), 3.14 (s, 6H), 2.82 (t, *J* = 7.6 Hz, 2H), 2.47 (m, 2H), 2.12 – 2.05 (m, 2H), 1.97 (m, 2H), 1.90 – 1.74 (m, 2H), 1.69 (m, 4H), 1.57 (m, 2H); ^{13}C NMR (101 MHz, $CDCl_3$) δ 163.8, 154.2, 151.7, 151.5, 140.9, 135.4, 133.2, 128.3, 125.3, 122.8, 122.7, 122.6, 121.4, 118.5, 118.2, 113.9, 100.8, 57.9, 57.1, 44.8, 30.6, 29.8, 29.7, 25.8, 24.9, 21.7; HRMS (ESI, m/z) calcd for $C_{27}H_{32}F_3N_6O_2S_2^+$ $[M + H]^+$, 593.1975, found 593.1972. HPLC purity 98%.

*1.9.6. 7-Cyclopentyl-2-((4-((5-isothiocyanatopentyl)sulfonyl)-2-(trifluoromethyl)phenyl)amino)-N,N-dimethyl-7H-pyrrolo[2,3-d]pyrimidine-6-carboxamide (**X6**).* Yield 60%; ^1H

NMR (400 MHz, $CDCl_3$) δ 9.03 (d, *J* = 8.9 Hz, 1H), 8.72 (s, 1H), 8.11 (d, *J* = 2.1 Hz, 1H), 7.99 (dd, *J* = 8.9, 2.2 Hz, 1H), 7.83 (s, 1H), 6.48 (s, 1H), 4.81 (m, 1H), 3.50 (t, *J* = 6.4 Hz, 2H), 3.13 (m, 8H), 2.50 – 2.41 (m, 2H), 2.14 – 2.06 (m, 2H), 2.01 – 1.95 (m, 2H), 1.83 – 1.76 (m, 2H), 1.70 (q, *J* = 7.4, 6.8 Hz, 4H), 1.54 (m, 2H); ^{13}C NMR (101 MHz, $CDCl_3$) δ 163.7, 153.6, 151.6, 151.2, 143.1, 133.6, 132.4, 130.3, 127.1, 127.0, 125.0, 122.3, 120.0, 117.4, 117.0, 114.3, 100.8, 58.0, 56.3, 44.7, 30.6, 29.7, 29.5, 25.4, 24.9, 22.2; HRMS (ESI, m/z) calcd for $C_{27}H_{32}F_3N_6O_3S_2^+$ [M + H]$^+$, 609.1924, found 609.1923. HPLC purity 99%.

*1.9.7. 7-Cyclopentyl-2-((4-((5-isothiocyanatopentyl)sulfinyl)phenyl)amino)-N,N-dimethyl-7H-pyrrolo[2,3-d]pyrimidine-6-carboxamide (**X7**).* Yield 27%; ^{1}H NMR (400 MHz, $CDCl_3$) δ 8.68 (s, 1H), 7.92 – 7.89 (m, 2H), 7.85 (s, 1H), 7.60 – 7.56 (m, 2H), 6.45 (s, 1H), 4.82 – 4.74 (m, 1H), 3.50 (t, *J* = 6.4 Hz, 2H), 3.15 (s, 6H), 2.82 (m, 2H), 2.61 – 2.55 (m, 2H), 2.11 – 2.03 (m, 4H), 1.77 – 1.67 (m, 6H), 1.59 – 1.51 (m, 2H); ^{13}C NMR (101 MHz, $CDCl_3$) δ 164.0, 154.8, 151.8, 151.6, 143.3, 135.0, 132.7, 125.3, 125.3, 118.6, 113.0, 101.0, 58.1, 57.0, 44.8, 39.5, 30.3, 29.7, 25.9, 24.8, 21.7; HRMS (ESI, m/z) calcd for $C_{26}H_{33}N_6O_2S_2^+$ [M + H]$^+$, 525.2101, found 525.2097. HPLC purity 99%.

*1.9.8. 7-Cyclopentyl-2-((4-((3-isothiocyanatopropyl)thio)phenyl)amino)-N,N-dimethyl-7H-pyrrolo[2,3-d]pyrimidine-6-carboxamide (**X8**).* Yield 41%; ^{1}H NMR (400 MHz, Chloroform-*d*) δ 8.65 (s, 1H), 7.71 – 7.66 (m, 2H), 7.41 – 7.35 (m, 3H), 6.43 (s, 1H),

4.78 (p, J = 8.9 Hz, 1H), 3.68 (t, J = 6.4 Hz, 2H), 3.15 (s, 6H), 2.96 (t, J = 6.8 Hz, 2H), 2.58 (t, J = 10.4 Hz, 2H), 2.11 – 2.02 (m, 4H), 1.98 – 1.92 (m, 2H), 1.75 – 1.67 (m, 2H); ^{13}C NMR (101 MHz, $CDCl_3$) δ 164.1, 155.2, 152.0, 151.7, 139.9, 136.1, 132.9, 132.2, 126.2, 119.2, 112.7, 101.1, 58.1, 43.6, 39.6, 32.6, 30.3, 29.5, 24.8; HRMS (ESI, m/z) calcd for $C_{24}H_{29}N_6OS_2^+$ $[M + H]^+$, 481.1839, found 481.1839. HPLC purity 98%.

*1.9.9. 7-Cyclopentyl-2-((4-((3-isothiocyanatopropyl)sulfonyl)phenyl)amino)-N,N-dimethyl-7H-pyrrolo[2,3-d]pyrimidine-6-carboxamide (**X9**).* Yield 34%; ^{1}H NMR (400 MHz, DMSO-d_6) δ 10.19 (s, 1H), 8.84 (s, 1H), 8.10 (t, J = 6.6 Hz, 2H), 7.79 (d, J = 8.6 Hz, 2H), 6.65 (s, 1H), 4.82 – 4.74 (m, 1H), 3.76 (t, J = 6.6 Hz, 2H), 3.34 (m, 2H), 3.06 (s, 6H), 2.45 (m, 2H), 2.00 (m, 4H), 1.92 (m, 2H), 1.68 (m, 2H); ^{13}C NMR (101 MHz, DMSO-d_6) δ 162.8, 154.6, 152.1, 150.9, 146.1, 132.6, 129.0, 128.8, 128.3, 117.4, 112.6, 100.6, 57.0, 52.4, 43.2, 34.6, 29.8, 24.2, 23.5; HRMS (ESI, m/z) calcd for $C_{24}H_{29}N_6O_3S_2^+$ $[M + H]^+$, 513.1737, found 513.1735. HPLC purity 96%.

*1.9.10. 7-Cyclopentyl-2-((4-((4-isothiocyanatobutyl)sulfonyl)phenyl)amino)-N,N-dimethyl-7H-pyrrolo[2,3-d]pyrimidine-6-carboxamide (**X10**).* Yield 53%; ^{1}H NMR (400 MHz, $CDCl_3$) δ 8.71 (s, 1H), 8.01 (d, J = 4.4 Hz, 1H), 7.96 – 7.92 (m, 2H), 7.83 (d, J = 8.9 Hz, 2H), 6.48 (s, 1H), 4.80 (t, J = 8.8 Hz, 1H), 3.54 (t, J = 6.0 Hz, 2H), 3.16 (s, 6H), 3.12 (d, J = 7.5 Hz, 2H), 2.61 – 2.53 (m, 2H), 2.13 – 2.04 (m, 4H), 1.90 – 1.81 (m, 4H); ^{13}C NMR (101 MHz, $CDCl_3$) δ 163.9, 154.4, 151.7, 151.6, 145.6, 133.1, 131.2,

130.0, 129.5, 117.7, 113.5, 100.9, 58.2, 55.7, 44.6, 39.5, 30.3, 28.6, 24.8, 20.5; HRMS (ESI, m/z) calcd for $C_{25}H_{31}N_6O_3S_2^+$ [M + H]$^+$, 527.1894, found 527.1895. HPLC purity 99%.

*1.9.11. 7-Cyclopentyl-2-((4-((5-isothiocyanatopentyl)sulfonyl)phenyl)amino)-N,N-dimethyl-7H-pyrrolo[2,3-d]pyrimidine-6-carboxamide (**X11**).* Yield 77%; ^{1}H NMR (400 MHz, $CDCl_3$) δ 8.71 (s, 1H), 7.98 (s, 1H), 7.93 (d, *J* = 8.9 Hz, 2H), 7.83 (d, *J* = 8.6 Hz, 2H), 6.47 (s, 1H), 4.78 (m, 1H), 3.50 (t, *J* = 6.4 Hz, 2H), 3.16 (s, 6H), 3.13 – 3.08 (m, 2H), 2.62 – 2.52 (m, 2H), 2.13 – 2.02 (m, 4H), 1.81 – 1.66 (m, 6H), 1.51 (m, 2H); ^{13}C NMR (101 MHz, $CDCl_3$) δ 163.9, 154.4, 151.7, 151.6, 145.5, 133.1, 130.4, 130.3, 129.5, 117.7, 113.5, 100.9, 58.2, 56.4, 44.8, 39.5, 30.3, 29.6, 25.5, 24.8, 22.4; HRMS (ESI, m/z) calcd for $C_{26}H_{33}N_6O_3S_2^+$ [M + H]$^+$, 541.2050, found 541.2049. HPLC purity 99%.

*1.9.12. 7-Cyclopentyl-2-((4-((6-isothiocyanatohexyl)sulfonyl)phenyl)amino)-N,N-dimethyl-7H-pyrrolo[2,3-d]pyrimidine-6-carboxamide (**X12**).* Yield 67%; ^{1}H NMR (400 MHz, $CDCl_3$) δ 8.70 (s, 1H), 7.99 (s, 1H), 7.94 – 7.91 (m, 2H), 7.83 (d, *J* = 8.8 Hz, 2H), 6.47 (s, 1H), 4.78 (m, 1H), 3.48 (t, *J* = 6.5 Hz, 2H), 3.15 (s, 6H), 3.11 – 3.07 (m, 2H), 2.62 – 2.53 (m, 2H), 2.13 – 2.02 (m, 4H), 1.73 (m, 4H), 1.68 – 1.62 (m, 2H), 1.41 (m, 4H); ^{13}C NMR (101 MHz, $CDCl_3$) δ 163.9, 154.4, 151.7, 151.6, 145.4, 133.1, 130.4, 130.0, 129.5, 117.7, 113.4, 100.9, 58.2, 56.5, 44.9, 39.5, 30.3, 29.7, 27.6, 26.2, 24.8,

22.8; HRMS (ESI, m/z) calcd for $C_{27}H_{35}N_6O_3S_2^+$ [M + H]$^+$, 555.2207, found 555.2207. HPLC purity 96%.

*1.9.13. 7-Cyclopentyl-2-((4-((7-isothiocyanatoheptyl)sulfonyl)phenyl)amino)-N,N-dimethyl-7H-pyrrolo[2,3-d]pyrimidine-6-carboxamide (**X13**).* Yield 51%; ^{1}H NMR (400 MHz, $CDCl_3$) δ 8.70 (s, 1H), 7.96 – 7.89 (m, 3H), 7.83 (d, *J* = 8.4 Hz, 2H), 6.47 (s, 1H), 4.79 (m, 1H), 3.47 (t, *J* = 6.7 Hz, 2H), 3.15 (s, 6H), 3.08 (t, *J* = 7.9 Hz, 2H), 2.57 (m, 2H), 2.07 (m, 4H), 1.73 (m, 5H), 1.64 (m, 2H), 1.38 (m, 4H), 1.33 – 1.28 (m, 2H); ^{13}C NMR (101 MHz, $CDCl_3$) δ 163.9, 154.4, 151.7, 151.6, 145.3, 133.1, 130.6, 129.9, 129.4, 117.7, 113.5, 100.9, 58.2, 56.6, 45.0, 39.5, 30.3, 29.9, 28.4, 28.2, 26.3, 24.8, 22.9; HRMS (ESI, m/z) calcd for $C_{28}H_{37}N_6O_3S_2^+$ [M + H]$^+$, 569.2363, found 569.2360. HPLC purity 96%.

*1.9.14. 7-Cyclopentyl-2-((4-(((3-isothiocyanatopropyl)thio)methyl)phenyl)amino)-N,N-dimethyl-7H-pyrrolo[2,3-d]pyrimidine-6-carboxamide (**X14**).* Yield 68%; ^{1}H NMR (400 MHz, $CDCl_3$) δ 8.63 (s, 1H), 7.69 – 7.65 (m, 2H), 7.41 (s, 1H), 7.29 – 7.26 (m, 2H), 6.43 (s, 1H), 4.81 – 4.75 (m, 1H), 3.71 (s, 2H), 3.61 (t, *J* = 6.4 Hz, 2H), 3.15 (s, 6H), 2.58 – 2.61 (m, 2H), 2.55 (t, *J* = 7.0 Hz, 2H), 2.09 – 2.02 (m, 4H), 1.89 (m, 2H), 1.71 (m, 2H); ^{13}C NMR (101 MHz, $CDCl_3$) δ 169.3, 164.2, 155.3, 152.2, 151.4, 139.3, 132.2, 131.2, 129.4, 118.9, 112.5, 101.1, 58.1, 43.9, 36.1, 30.3, 29.8, 29.5, 28.1, 24.8; HRMS (ESI, m/z) calcd for $C_{25}H_{31}N_6OS_2^+$ [M + H]$^+$, 495.1995, found 495.1998. HPLC purity 95%.

1.9.15. 7-Cyclopentyl-2-((4-(((4-isothiocyanatobutyl)thio)methyl)phenyl)amino)-N,N-dimethyl-7H-pyrrolo[2,3-d]pyrimidine-6-carboxamide ***(X15)***. Yield 18%; ^{1}H NMR (400 MHz, $CDCl_3$) δ 8.65 (s, 1H), 7.69 (d, *J* = 8.8 Hz, 2H), 7.46 (s, 1H), 7.28 (d, *J* = 8.4 Hz, 2H), 6.41 (s, 1H), 4.79 (m, 1H), 3.74 (t, *J* = 7.8 Hz, 2H), 3.49 (t, *J* = 6.7 Hz, 2H), 3.16 (s, 6H), 2.59 (m, 2H), 2.46 (t, *J* = 7.2 Hz, 2H), 2.07 (m, 4H), 1.72 (m, 6H); ^{13}C NMR (101 MHz, $CDCl_3$) δ 164.2, 155.5, 152.3, 151.6, 139.3, 132.2, 131.5, 130.7, 129.4, 118.9, 112.5, 101.1, 58.1, 44.8, 39.6, 36.0, 30.4, 30.4, 29.1, 26.2, 24.8; HRMS (ESI, m/z) calcd for $C_{26}H_{33}N_6OS_2^+$ $[M + H]^+$, 509.2152, found 509.2156. HPLC purity 98%.

1.9.16. 7-Cyclopentyl-2-((4-(((5-isothiocyanatopentyl)thio)methyl)phenyl)amino)-N,N-dimethyl-7H-pyrrolo[2,3-d]pyrimidine-6-carboxamide ***(X16)***. Yield 27%; ^{1}H NMR (400 MHz, $CDCl_3$) δ 8.65 (s, 1H), 7.67 (d, *J* = 8.5 Hz, 2H), 7.44 (s, 1H), 7.28 (d, *J* = 8.5 Hz, 2H), 6.43 (s, 1H), 4.83 – 4.73 (m, 1H), 3.71 (s, 2H), 3.49 (t, *J* = 6.6 Hz, 2H), 3.16 (s, 6H), 2.65 – 2.55 (m, 2H), 2.45 (t, *J* = 7.2 Hz, 2H), 2.12 – 2.00 (m, 4H), 1.75 – 1.63 (m, 4H), 1.64 – 1.53 (m, 2H), 1.54 – 1.43 (m, 2H); ^{13}C NMR (101 MHz, $CDCl_3$) δ 164.2, 155.5, 152.2, 151.7, 139.2, 132.0, 131.6, 129.4, 129.4, 118.7, 112.5, 101.1, 58.0, 45.0, 39.5, 36.1, 31.0, 30.3, 29.7, 28.5, 25.9, 24.8; HRMS (ESI, m/z) calcd for $C_{27}H_{35}N_6OS_2^+$ $[M + H]^+$, 523.2308, found 523.2310. HPLC purity 99%.

1.9.17. 7-cyclopentyl-2-((4-(((6-isothiocyanatohexyl)thio)methyl)phenyl)amino)-N,N-dimethy

l-7H-pyrrolo[2,3-d]pyrimidine-6-carboxamide ***(X17)***. Yield 35%; ^{1}H NMR (400 MHz, $CDCl_3$) δ 8.65 (s, 1H), 7.67 (d, *J* = 8.2 Hz, 2H), 7.46 (s, 1H), 7.27 (d, *J* = 8.5 Hz, 2H), 6.43 (s, 1H), 4.79 (m, 1H), 3.71 (s, 2H), 3.48 (t, *J* = 6.6 Hz, 2H), 3.16 (s, 6H), 2.64 – 2.56 (m, 2H), 2.44 (t, *J* = 7.3 Hz, 2H), 2.06 (m, 4H), 1.73 – 1.65 (m, 4H), 1.58 (m, 2H), 1.41 – 1.36 (m, 4H); ^{13}C NMR (101 MHz, $CDCl_3$) δ 164.2, 155.5, 152.2, 151.7, 139.2, 132.9, 132.0, 131.7, 129.3, 118.7, 112.4, 101.1, 58.0, 45.1, 39.6, 36.0, 31.2, 30.3, 29.9, 29.0, 28.1, 26.3, 24.8; HRMS (ESI, m/z) calcd for $C_{28}H_{37}N_6OS_2^+$ [M + H]$^+$, 537.2465, found 537.2466. HPLC purity 99%.

1.9.18. 7-Cyclopentyl-2-((4-(((7-isothiocyanatoheptyl)thio)methyl)phenyl)amino)-N,N-dimethyl-7H-pyrrolo[2,3-d]pyrimidine-6-carboxamide ***(X18)***. Yield 37%; ^{1}H NMR (400 MHz, $CDCl_3$) δ 8.57 (s, 1H), 7.63 – 7.51 (m, 3H), 7.20 (d, *J* = 7.7 Hz, 2H), 6.36 (s, 1H), 4.71 m, 1H), 3.63 (s, 2H), 3.40 (t, *J* = 6.6 Hz, 2H), 3.08 (s, 6H), 2.52 (m, 2H), 2.36 (t, *J* = 7.3 Hz, 2H), 1.98 (m, 4H), 1.61 (m, 4H), 1.53 – 1.46 (m, 2H), 1.29 (m, 6H); ^{13}C NMR (101 MHz, $CDCl_3$) δ 164.2, 155.5, 152.2, 151.6, 139.1, 132.0, 131.8, 130.6, 129.3, 118.7, 112.4, 101.1, 58.0, 45.1, 36.0, 31.3, 30.3, 29.9, 29.8, 29.1, 28.7, 28.5, 26.5, 24.8; HRMS (ESI, m/z) calcd for $C_{29}H_{39}N_6OS_2^+$ [M + H]$^+$, 551.2621, found 551.2620. HPLC purity 96%.

1.9.19. 7-Cyclopentyl-2-((4-(((3-isothiocyanatopropyl)sulfonyl)methyl)phenyl)amino)-N,N-dimethyl-7H-pyrrolo[2,3-d]pyrimidine-6-carboxamide ***(X19)***. Yield 70%; ^{1}H NMR (400 MHz, $CDCl_3$) δ 8.67 (s, 1H), 7.78 (d, *J* = 8.3 Hz, 2H), 7.54 (s, 1H), 7.36 (d, *J* =

8.8 Hz, 2H), 6.44 (s, 1H), 4.92 – 4.73 (m, 1H), 4.40 – 4.20 (m, 2H), 3.80 – 3.62 (m, 2H), 3.15 (s, 6H), 3.08 – 2.91 (m, 2H), 2.57 (m, 2H), 2.23 – 2.00 (m, 6H), 1.71 (m, 2H); ^{13}C NMR (101 MHz, $CDCl_3$) δ 164.2, 152.1, 151.7, 141.5, 141.5, 133.1, 132.5, 131.2, 120.3, 119.0, 113.0, 101.0, 60.2, 58.1, 47.8, 43.9, 39.5, 30.4, 24.9, 22.8; HRMS (ESI, m/z) calcd for $C_{25}H_{31}N_6O_3S_2^+$ [M + H]$^+$, 527.1894, found 527.1892. HPLC purity 98%.

*1.9.20. 7-Cyclopentyl-2-((4-(((4-isothiocyanatobutyl)sulfonyl)methyl)phenyl)amino)-N,N-dimethyl-7H-pyrrolo[2,3-d]pyrimidine-6-carboxamide (**X20**).* Yield 50%; ^{1}H NMR (400 MHz, $CDCl_3$) δ 8.67 (s, 1H), 7.78 (d, J = 8.6 Hz, 2H), 7.49 (s, 1H), 7.37 (d, J = 8.7 Hz, 2H), 6.44 (s, 1H), 4.79 (m, 1H), 4.24 (s, 2H), 3.54 (t, J = 6.3 Hz, 2H), 3.16 (s, 6H), 2.88 (t, J = 7.6 Hz, 2H), 2.62 – 2.52 (m, 2H), 2.06 (m, 4H), 1.99 – 1.90 (m, 2H), 1.82 (m, 2H), 1.71 (m, 2H); ^{13}C NMR (101 MHz, $CDCl_3$) δ 164.1, 155.1, 152.0, 151.7, 144.6, 141.3, 132.4, 131.1, 120.5, 118.9, 112.9, 101.1, 59.7, 58.0, 49.7, 44.6, 39.6, 30.4, 28.8, 24.8, 19.4; HRMS (ESI, m/z) calcd for $C_{26}H_{33}N_6O_3S_2^+$ [M + H]$^+$, 541.2050, found 541.2050. HPLC purity 97%.

*1.9.21. 7-Cyclopentyl-2-((4-(((5-isothiocyanatopentyl)sulfonyl)methyl)phenyl)amino)-N,N-dimethyl-7H-pyrrolo[2,3-d]pyrimidine-6-carboxamide (**X21**).* Yield 74%; ^{1}H NMR (400 MHz, $CDCl_3$) δ 8.67 (s, 1H), 7.77 (d, J = 8.6 Hz, 2H), 7.59 (s, 1H), 7.35 (d, J = 8.3 Hz, 2H), 6.44 (s, 1H), 4.80 (m, 1H), 4.22 (s, 2H), 3.50 (t, J = 6.4 Hz, 2H), 3.15 (s, 6H), 2.88 – 2.83 (m, 2H), 2.57 (m, 2H), 2.11 – 2.00 (m, 4H), 1.84 (m, 2H), 1.75 –

1.65 (m, 4H), 1.56 – 1.47 (m, 2H); ^{13}C NMR (101 MHz, $CDCl_3$) δ 164.1, 155.2, 152.0, 151.8, 144.8, 141.3, 132.3, 131.1, 120.5, 118.8, 112.8, 101.0, 59.6, 58.0, 50.5, 44.7, 39.6, 30.3, 29.6, 25.6, 24.8, 21.1; HRMS (ESI, m/z) calcd for $C_{27}H_{35}N_6O_3S_2^+$ [M + H]$^+$, 555.2207, found 555.2208. HPLC purity 99%.

*1.9.22. 7-Cyclopentyl-2-((4-(((6-isothiocyanatohexyl)sulfonyl)methyl)phenyl)amino)-N,N-dimethyl-7H-pyrrolo[2,3-d]pyrimidine-6-carboxamide (**X22**).* Yield 67%; ^{1}H NMR (400 MHz, $CDCl_3$) δ 8.66 (s, 1H), 7.77 (d, *J* = 8.6 Hz, 2H), 7.47 (s, 1H), 7.36 (d, *J* = 8.7 Hz, 2H), 6.44 (s, 1H), 4.80 (m, 1H), 4.21 (s, 2H), 3.49 (t, *J* = 6.5 Hz, 2H), 3.15 (s, 6H), 2.87 – 2.82 (m, 2H), 2.62 – 2.53 (m, 2H), 2.11 – 2.01 (m, 4H), 1.86 – 1.78 (m, 4H), 1.73 – 1.64 (m, 4H), 1.43 (m, 4H); ^{13}C NMR (101 MHz, $CDCl_3$) δ 164.1, 155.1, 152.0, 151.7, 141.2, 132.4, 131.1, 127.9, 120.7, 118.8, 112.8, 101.1, 59.5, 58.0, 50.6, 45.0, 39.5, 30.3, 29.6, 27.8, 26.2, 24.8, 21.6; HRMS (ESI, m/z) calcd for $C_{28}H_{37}N_6O_3S_2^+$ [M + H]$^+$, 569.2363, found 569.2345. HPLC purity 99%.

*1.9.23. 7-Cyclopentyl-2-((4-(((7-isothiocyanatoheptyl)sulfonyl)methyl)phenyl)amino)-N,N-dimethyl-7H-pyrrolo[2,3-d]pyrimidine-6-carboxamide (**X23**).* Yield 51%; ^{1}H NMR (400 MHz, DMSO-d_6) δ 9.67 (s, 1H), 8.76 (s, 1H), 7.86 (d, *J* = 8.3 Hz, 2H), 7.31 (d, *J* = 8.3 Hz, 2H), 6.59 (s, 1H), 4.74 (m, 1H), 4.38 (s, 2H), 3.62 (t, *J* = 6.7 Hz, 2H), 3.10 – 2.99 (m, 8H), 2.47 (m, 2H), 1.98 (m, 4H), 1.63 (m, 6H), 1.31 (m, 6H); ^{13}C NMR (101 MHz, DMSO-d_6) δ 162.9, 155.3, 152.1, 151.1, 141.2, 131.8, 131.1, 127.2, 120.6, 118.0, 111.7, 100.7, 57.6, 57.0, 50.5, 44.6, 34.6, 29.6, 29.1, 27.7, 27.6, 25.7, 24.2,

21.1; HRMS (ESI, m/z) calcd for $C_{29}H_{39}N_6O_3S_2^+$ $[M + H]^+$, 583.2520, found 583.2520.

HPLC purity 98%.

Copy of 1H- and 13C-NMR Spectra for Compound X22

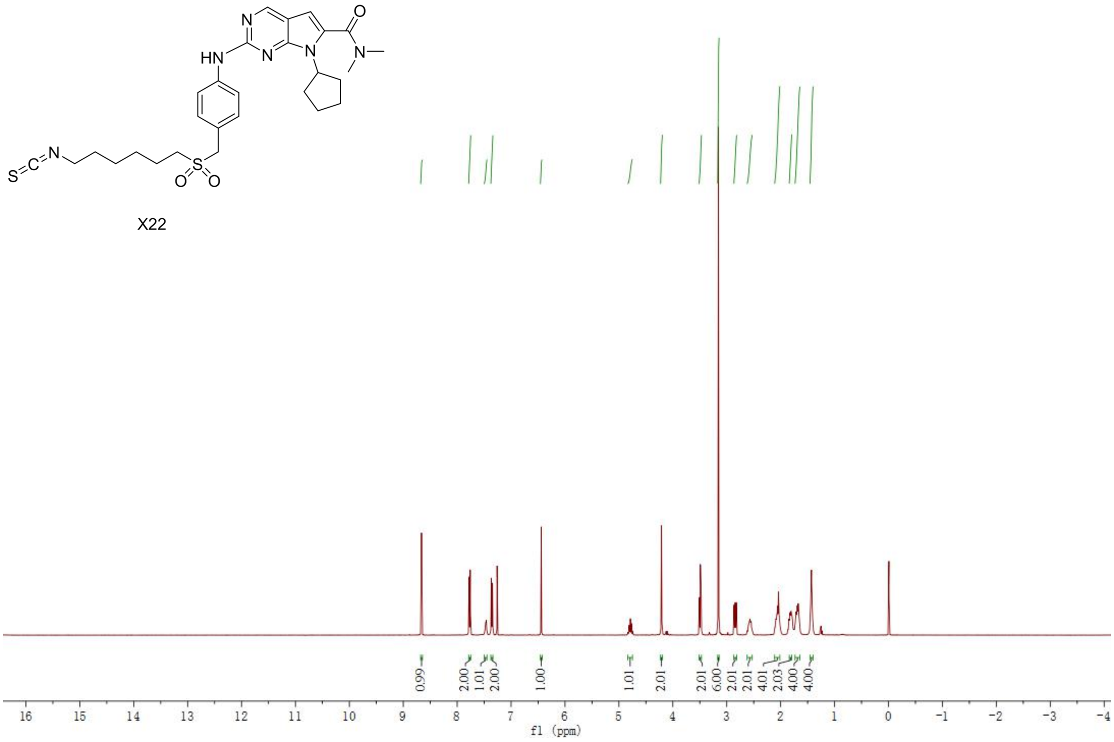

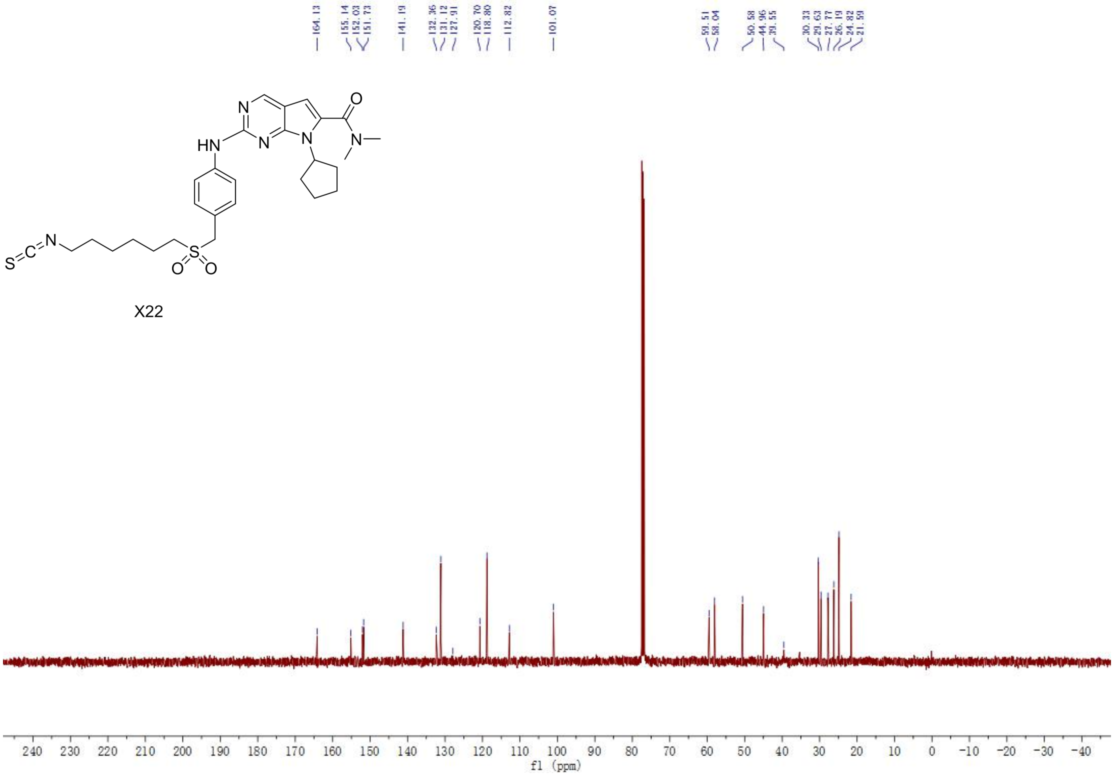

Copy of HRMS Spectra for Compound X22

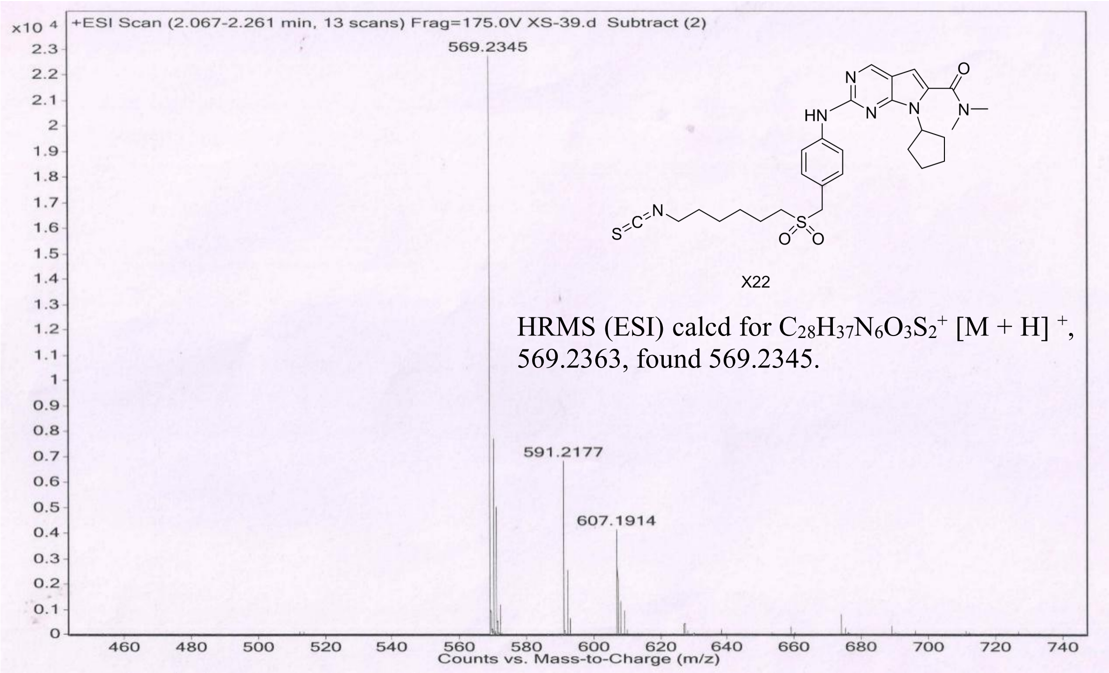

HPLC purity analysis for Compound X22

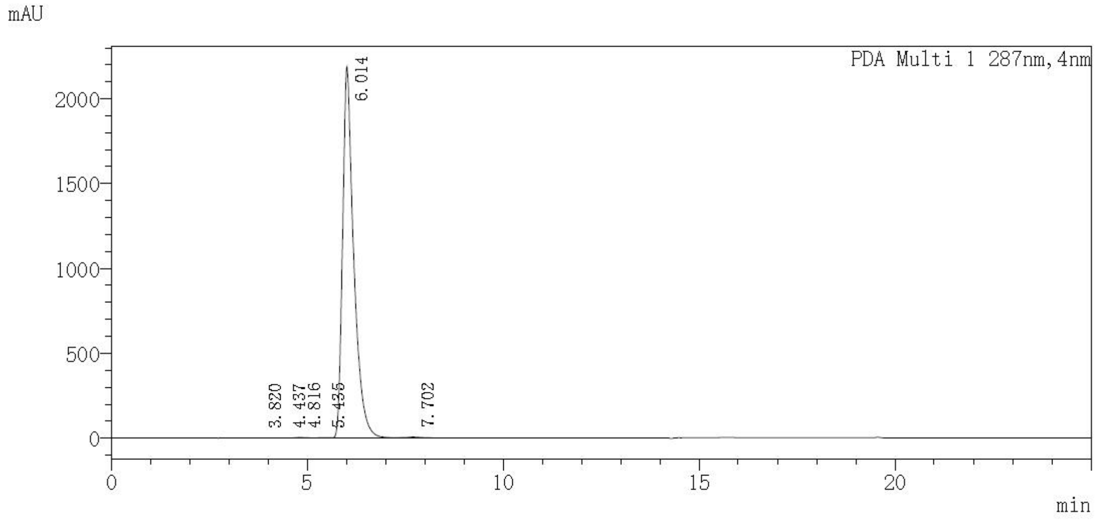

PDA Ch2 287nm

Peak NO.	RT(min)	Area	Area%
1	3.820	4900	0.011
2	4.437	5732	0.013
3	4.816	56043	0.129
4	5.435	40037	0.092
5	6.014	43328069	99.489
6	7.702	115754	0.266
Total		43550536	100.000
